# Lack of extracellular matrix switches TGF-β induced apoptosis of endometrial cells to epithelial to mesenchymal transition

**DOI:** 10.1038/s41598-022-18976-1

**Published:** 2022-09-01

**Authors:** Anna Ruiz-Mitjana, Raúl Navaridas, Maria Vidal-Sabanés, Aida Perramon-Güell, Andree Yeramian, Isidre Felip, Núria Eritja, Joaquim Egea, Mario Encinas, Xavier Matias-Guiu, Xavier Dolcet

**Affiliations:** 1 Oncologic Pathology Group, Departament de Ciències Mèdiques Bàsiques, Universitat de Lleida, Institut de Recerca Biomèdica de Lleida, IRBLleida, Ed Biomedicina I, Hospital Arnau de Vilanova, Av Rovira Roure, 80, 25198 Lleida, Spain; 2 Molecular Developmental Neurobiology Group, Departament de Ciències Mèdiques Bàsiques, Universitat de Lleida, Institut de Recerca Biomèdica de Lleida, IRBLleida, Lleida, Spain; 3 Developmental and Oncogenic Signaling Group, Departament de Medicina Experimental, Universitat de Lleida, Institut de Recerca Biomèdica de Lleida, IRBLleida, Lleida, Spain

**Keywords:** Cancer, Cell biology

## Abstract

The extracellular matrix and the correct establishment of epithelial cell polarity plays a critical role in epithelial cell homeostasis and cell polarity. In addition, loss of tissue structure is a hallmark of carcinogenesis. In this study, we have addressed the role of extracellular matrix in the cellular responses to TGF-β. It is well known that TGF-β is a double-edged sword: it acts as a tumor suppressor in normal epithelial cells, but conversely has tumor-promoting effects in tumoral cells. However, the factors that determine cellular outcome in response to TGF-β remain controversial. Here, we have demonstrated that the lack of extracellular matrix and consequent loss of cell polarity inhibits TGF-β-induced apoptosis, observed when endometrial epithelial cells are polarized in presence of extracellular matrix. Rather, in absence of extracellular matrix, TGF-β-treated endometrial epithelial cells display features of epithelial-to-mesenchymal transition. We have also investigated the molecular mechanism of such a switch in cellular response. On the one hand, we found that the lack of Matrigel results in increased AKT signaling which is sufficient to inhibit TGF-β-induced apoptosis. On the other hand, we demonstrate that TGF-β-induced epithelial-to-mesenchymal transition requires ERK and SMAD2/3 activation. In summary, we demonstrate that loss of cell polarity changes the pro-apoptotic function of TGF-β to tumor-associated phenotype such as epithelial-to-mesenchymal transition. These results may be important for understanding the dual role of TGF-β in normal versus tumoral cells.

## Introduction

Cell-to-cell, cell-to extracellular matrix (ECM) interactions and establishment of a correct cell polarity play a pivotal role in epithelial cell homeostasis. Mounting evidences support that the three dimensional (3D) structure of epithelial tissue plays an important role in deciding cell fate and is involved in the regulation of proliferation, survival and apoptosis of individual epithelial cells^1–3^. Cell polarity acts as a tumor suppressor mechanism and its loss is a hallmark of cancer^2^.

Over the past decades, 3D organoid cultures derived from epithelial tissues have been developed to recreate structures that reassemble their in vivo architecture^4^. Such culture models have emerged as powerful cell-based scenarios to investigate many aspects of cancer pathobiology, such as the role of cell polarity and ECM, the function of tumor suppressors or oncogenes, and the importance of pathways in cancer^5^. To investigate the pathobiology of cancer it is biologically relevant to maintain the features of the 3D architecture of the epithelial tissue in culture. It is known that significant differences are observed between experiments assessed with cells cultured in a 3D polarity structures and experiments using classical two-dimensional (2D) cultures. Consequently, it has been proposed 3D polarity as a non-canonical mechanism of tumor suppression since it may function as an inhibitor of primary tumor development^6^.

In normal epithelial tissues, Transforming Growth Factor-β (TGF-β) regulates a plethora of cell functions and processes depending on cell type, cellular context and microenvironment^7–9^. TGF-β is secreted as an inactive latent disulfide-linked homodimer (LTGF-β) polypeptide. Mature bioactive TGF-β ligands are produced upon proteolytic cleavage of the latent complex and directly bind to TGF-β type II receptor (TGF-βRII). TGF-βRII phosphorylates and activates the TGF-β receptor type I which, in turn, phosphorylates members of the SMAD family^10^. The SMAD family consists of nine members, which form 3 subfamilies: receptor-activated (R-)SMADs (SMAD1, SMAD2, SMAD3, SMAD5, SMAD8 and SMAD9), a single common-mediator (Co-)SMAD (SMAD4) and two inhibitory (I-)SMADs (SMAD6 and SMAD7). TGF-β also induces non-SMAD pathways, including mitogen-activated protein kinase (MAPK), phosphoinositide-3-kinase (PI3K) and Rho GTPases^11^.

Dysregulation of TGF-β signaling has been implicated in the progression of diseases such as fibrosis or cancer. In most normal epithelial tissues, TGF-β acts as tumor suppressor by inhibiting cell cycle progression or activating apoptosis but, during carcinogenesis, TGF-β switches its tumor suppressive functions to promoter ones^12–15^. However, the mechanisms of these functional changes of TGF-β are not fully understood and may vary among cell types and contexts. As a tumor promoter, TGF-β stimulation is associated with epithelial-to-mesenchymal transition (EMT) and acquisition of cell migration, invasion and metastatic potential of malignant epithelial cells^14,15^.

EMT is a well-coordinated process during embryonic development and a pathological feature in neoplasia and fibrosis. Cells undergoing EMT lose the expression of epithelial cell markers such as cytokeratin, E-cadherin or β-catenin and gain the expression of mesenchymal markers such as vimentin^16^. However, over the past few years, numerous studies demonstrate that EMT is a dynamic process in which cells undergoing partial or hybrid EMT display intermediate cell states retaining expression of both epithelial and mesenchymal markers^17–19^. Although EMT has traditionally been associated with increased metastatic properties, recent studies have activated the debate on the requirement of EMT and its different EMT states for cancer metastasis^17–19^. To this regard, a dynamic process designated as partial or hybrid EMT in which cells display markers and features of both epithelial and mesenchymal phenotype has been demonstrated to be important in cancer metastasis and therapy resistance^19,46,47^.

Here, using a 3D organoid culture system of mouse endometrial epithelial cells, we have investigated the effect of extracellular matrix-induced 3D cell organization on biological responses to TGF-β. We have demonstrated that the lack of ECM and subsequent loss of cell polarity switches TGF-β-induced apoptosis to an EMT-compatible phenotype. We have also inferred the molecular mechanism involved in this swapped function. On the one hand, we demonstrated that the absence of ECM controls sensitivity to TGF-β-induced apoptosis through regulation of AKT signaling. On the other hand, both ERK and SMAD2/3 signaling regulate TGF-β-triggered EMT in absence of ECM. In summary, our results support that ECM-induced cell polarity acts as a non-canonical tumor suppressor for endometrial epithelial cells.

## Material and methods

### Reagents and antibodies

The recombinant basement membrane Matrigel was purchased from BD Biosciences (San Jose, CA). Epidermal Growth Factor, hydrocortisone, dexamethasone, RU-486, ICI 182170 and LY 294002 were obtained from Sigma (St Louis, MO), Insulin–Transferrin–Sodium Selenite supplement was obtained from Invitrogen (Invitrogen, Inc., Carlsbad, CA, USA), PD0325901 and U0126 were purchased from Calbiochem (Calbiochem-Novabiochem, UK, Ltd). Antibodies to E-Cadherin (#610181), β-Catenin (#610153), panERK (#610623) and Vimentin (#550513) were from BD Biosciences; bisBenzimide H 33342 trihydrochloride (Hoechst), anti-β-actin (#a5441) and tubulin (T6199) were obtained from Sigma and anti-cytokeratin (#9377) was from Abcam (Cambridge UK). Anti-phospho-AKT serine 473 (#9271), anti-phospho-p70SK6 threonine 389 (#9205), cleaved caspase-3 (#9661), PTEN (#9188) and phospho-mTOR serine 2448 (#2971) antibodies were from Cell Signaling Technology (Beverly, MA). Anti-phospho-FAK 397 (#700255) was from Thermo fisher. Anti-phospho-ERK threonine 202/204 (#22.675505) was from Biolegend. Anti-AKT (sc-1618) and anti-phospho-Smad2/3 serine 423/425 (sc -11769) were from Santa Cruz Biotechnology, Inc. Alexa-Fluor anti-Rabbit (#11037) and anti-mouse (#11029) antibodies were from Invitrogen. Peroxidase-conjugated anti-mouse (#115-007-003) and anti-rabbit (#011-000-003) antibodies were from Jackson ImmunoResearch Europe Ltd (Suffolk, UK). PD184352 (CI-1040), Mirdametinib (PD0325901) and Pictilisib (GDC-0941) were purchased from Selleckchem (Selleck Chemicals LLC).

All other regents were obtained from Sigma unless specified.

### Animals and Isolation of endometrial epithelial cells

Mouse were housed, maintained, breed and genotyped as previously described was performed as previously described^21,22^. Mice were housed in a barrier facility and pathogen-free procedures were used in all mouse rooms. Animals were kept in a 12-h light-dark cycle with ad libitum access to a standard 2014 Teklad Global 14% Protein Rodent Maintenance Diet (www.harlan.com) and water. The animal rooms were environmentally controlled (20 ± 2 °C, relative humidity 50 ± 5%). The study complied with Law 5/1995 and Act 214/1997 of the Autonomous Community (Generalitat of Catalonia) and EU Directive EEC 63/2010, and was approved by the Ethics Committee on Animal Experiments of the University of Lleida and the Ethics Commission in Animal Experimentation of the Generalitat de Catalunya.

CRE:ER (B6.Cg-Tg(CAG-CRE/Esr1* 5Amc/J) mice was obtained from the Jackson Laboratory (Bar Harbor, ME, USA). Double Smad2^f/f^ Smad3^f/f^ mice was provided by Dr.Martin M Matzuk (Department of Pathology, Baylor College of Medicine, One Baylor Plaza, Houston, Texas, USA).

CRE:ER mice genotyping PCR was carried out with the following primer: forward primer 5′-CAC TCC CAG AGA CAT ATA CAC-3′ and reverse primer 5′-ACG AAC CTG GTC GAA ATC GT GCG-3′.

Smad3^f/f^ mice genotyping PCR was carried out with the following primer: forward primer 5′-CTC CAG ATC GTG GGC ATA CAG C-3′; *Smad3*^f/f^ reverse primer 5′-GGT CAC AGG GTC CTC TGT GCC-3′.

Smad2^f/f^ mice genotyping PCR was carried out with the following primer: forward primer 5′-CAT CAG ATT CCA TTA GAG ATG G-3′; *Smad2*^f/f^ reverse primer 5′-TGA GAC TTC TCT GTA CCC GAT-3′.

CRE:ER^+/−^ Smad2^f/f^ Smad3^f/f^ mice were bred in a mixed background (C57BL6; 129S4) by crossing Smad2^f/f^ Smad3^f/f^ mice with CRE:ER^+/−^ mice. To obtain mice carrying both Smad2 and Smad3 floxed alleles and a single CRE:ER (CRE:ER^+/−^), CRE:ER^+/−^ were backcrossed with Smad2^f/f^; Smad2^f/f^ mice. The isolation of endometrial epithelial cells was processed as described previously^20^. Briefly, uterine horns were dissected from 3 to 4 weeks old C57BL6 mice. Uterus were washed with Hank’s Buffered Saline Solution (HBSS) and digested with Trypsin (Invitrogen). After trypsin digestion epithelial sheets were squeezed-out of the uterine pieces. Epithelial sheets were washed twice with PBS and resuspended in 1 ml of DMEM/F12 (Invitrogen) supplemented with 1% of Sodium Pyruvate (Sigma), 1% of penicillin/streptomycin (Sigma) and 0.1% of fungizone (Invitrogen) (basal medium). Epithelial sheets were mechanically disrupted in basal medium until small clumps of cells were observed.

### 2D endometrial monolayers and 3D endometrial organoids cultures

Growth of endometrial epithelial cells in 2D cultures was performed by platting cells in basal medium with 2% of dextran-coated charcoal-stripped serum (DCC) (Invitrogen) and leaved twenty-four hours. To induce deletion of floxed alleles, Tamoxifen (Sigma-Aldrich T5648, St. Louis, MO, USA) was dissolved in 100% EtOH at 1 mM and used at 0.5 μl/ml of medium for 24 h. For immunofluorescence, cells were seeded in a volume of 40 μl/well in 96 well plates black with micro-clear bottom (Greiner Bio-one). For western blotting, cells were placed in a volume of 200 μl in 24-well plates (BD Biosciences).

Growth of endometrial epithelial cells in 3D cultures was performed as described previously^20^. Twenty-four hours after platting in plastic, cells were washed with HBSS and incubated with trypsin/EDTA solution (Sigma) for 5 min at 37 °C. Trypsin was stopped by adding DMEM 10% FBS and clumps of 2–8 cells were obtained. Cells were centrifuged at 1000 rpm for 3 min and diluted in basal medium containing 3% of Matrigel to obtain 4 × 10^4^ cell clumps/ml. For immunofluorescence, cells were seeded in a volume of 40 μl/well in 96 well plates (black with micro-clear bottom) (Greiner Bio-one). For western blotting, cells were placed in a volume of 200 μl in 24-well plates (BD Biosciences). In all cases, 24 h after plating medium was replaced by Basal medium supplemented with 5 ng/ml EGF and 1/100 dilution of Insulin–Transferrin–Sodium Selenite (ITS) Supplement (Invitrogen) and 3% of fresh Matrigel (referred as BIE). Medium was replaced every 2–3 days.

### Immunofluorescence

Immunofluorescence was performed as previously described^20,21^. Cultures were fixed with formalin for 5 min at room temperature (RT) and washed twice with PBS. Depending on primary antibody, cells were permeabilized with 0.2% triton X-100 in PBS for 10 min (indicated as T) or permeabilized with 100% methanol for 2 min (indicated as M). Next, cultures were incubated overnight at 4 °C with the indicated dilutions of antibodies: anti-Cytokeratin 1/500 (M), β-Catenin 1/100 (M), E-Cadherin 1/100 (M), Rhodamine conjugated-Phalloidin 1/500 (T), anti-Cleaved Caspase-3 1/100 (T), anti-Vimentin 1/100 (T) and anti-phospho-FAK 1/100 (T). After one day, cells were washed twice with PBS and incubated with PBS containing a 5 μg/ml of Hoechst dye and 1/500 dilution of Alexa Fluor secondary anti-mouse or anti-rabbit antibodies for 2 h at RT in the case of 2D cultures or overnight at 4 °C for 3D cultures. For double immunofluorescence staining, cells were incubated with the second round of primary and secondary antibodies. We would like to point out that in all double immunofluorescence stains, first and second primary antibodies were from different isotope. Immunofluorescence staining was visualized and analyzed using a confocal microscopy (Olympus). Edition of confocal images was performed using Fluoview software (Olympus).

For evaluation of apoptosis and caspase-3 processing, endometrial epithelial glands were analyzed on a confocal microscope Fluoview FV1000. The presence of apoptotic or positive caspase-3 processing (more than 5 cells per gland) was revealed by Hoechst/Cleaved Caspase-3 immunostaining. For each experiment we quantified at least 100 glands. Cell polarity of epithelial cells forming glandular structures was evidenced by double immunostainings as indicated in each figure.

### Western blot analysis

Western blot was performed as previously described^21,22^. Briefly, 2D endometrial monolayers or endometrial organoid 3D cultures stimulated for the indicated periods of time, were washed with HBSS and incubated with trypsin/EDTA solution for 5 min at 37 °C. Incubation with trypsin was done to allow us to separate the glandular structures from Matrigel. Trypsin was stopped by adding DMEM 10% FBS and the cells were lysed with lysis buffer (2% SDS, 125 mM Tris–HCL pH6.8). Relative protein concentrations were determined loading an 8% acrylamide gel, transferred to PVDF membranes and blotted with anti-tubulin antibody. Equal amounts of proteins were subjected to SDS-PAGE and transferred to PVDF membranes (Millipore, Bedford, MA). Non-specific binding was blocked by incubation with TBST (20 mM Tris–Hcl pH7.4, 150 mM NaCl, 0.1% Tween-20) plus 5% of non-fat milk. Membranes were incubated overnight at 4 °C with the indicated dilution of antibodies: PTEN 1/1000, pERK 1/1000, panERK 1/1000, pAKT 1/1000, AKT Total 1/100, p-p70S6K 1/1000, E-cadherin 1/1000, β-catenin 1/1000, vimentin 1/1000, p-Mtor 1/1000 and tubulin 1/10,000. The procedure was followed by 1-h incubation with secondary antibody 1/10,000 in TBST at RT. Signal was detected with Immobilon Forte Western HRP Substrate (EMD Millipore Corporation, Burlington).

### Real-time PCR

RNA extraction and real-time PCR was performed as previously described^21^.Total RNA was prepared using NZYol from Nzytech according to the manufacturer’s protocol. Reverse transcription reaction was performed using a total 1 μg total RNA with TaqMan® Reverse Transcription Kit from Applied Biosystems. Quantitative real-time PCR detection of gene expression was performed with the ABI Prism 7000 Sequence Detection System using the TaqMan® Universal PCR Master Mix (Applied Biosystems). The sequences of primers used for PCR were obtained commercially from Applied biosystems Assay-on-demand Gene Vimentin (Mm01333430_m1), Gene Cdh2 (Mm01162497_m1), Gene Twist1 (Mm00442036_m1) and Gadph (Mm99999915_g1). Relative expression was determined from cycle threshold (Ct) values, which were normalized to Gadph as the endogenous control. Experiments were performed at least three times and statistical significance was determined by student’s *t* test with *p* value ≤ 0.05.

### Transwell migration assay

Cells were seeded in basal medium on top of 8 µm Transwell inserts (Falcon) in presence or absence of tamoxifen (+ /− TAM) and left to attach for 24 h and then, medium was changed for basal medium alone or basal medium supplemented with TGF-β. After 72 h, medium was removed, and the upper side of the filter membranes was gently cleaned with a cotton swab. Membranes were cut out from the inserts with a razor blade, and the cells from the bottom side of the membrane (migrating cells) were stained with 1% Hoechst (Sigma) for 10 min at room temperature. Nuclei were scored and analyzed under epifuorescence microscope. Ten random fields were examined, and the results were plotted as the percentage of control. The experiments were repeated three times, and every treatment group was performed in triplicate.

### Statistical analysis

Statistical analysis was performed as previously described with the appropriate modifications^21,22^. Experiments were performed at least three times and statistical significance was determined by student’s test with *p* value ≤ 0.01. Unless otherwise asterisks indicate different *p* value. Statistical analysis was performed with GraphPad Prism 8.0. Differences between two groups were assessed by Student’s *t* test (unpaired or paired as needed depending on the study design). Differences between more than two groups were assessed by one-way ANOVA, followed by the Tukey’s multiple comparison test or two-way ANOVA, followed by the Bonferroni post hoc comparison test. A *p* value ≤ 0.05 was considered statistically significant. All data examined are expressed as mean ± SEM.

### Ethics approval

For animal studies, we complied with Law 5/1995 and Act 214/1997 of the Autonomous Community (Generalitat of Catalonia) and EU Directive EEC 63/2010, and was approved by the Ethics Committee on Animal Experiments of the University of Lleida and the Ethics Commission in Animal Experimentation of the Generalitat de Catalunya. *Arrive Statement*: All animal procedures were performed in accordance with ARRIVE guidelines.

## Results

### Loss of cell polarity caused by absence of ECM impairs TGF-β-induced apoptosis.

**Figure 1:**
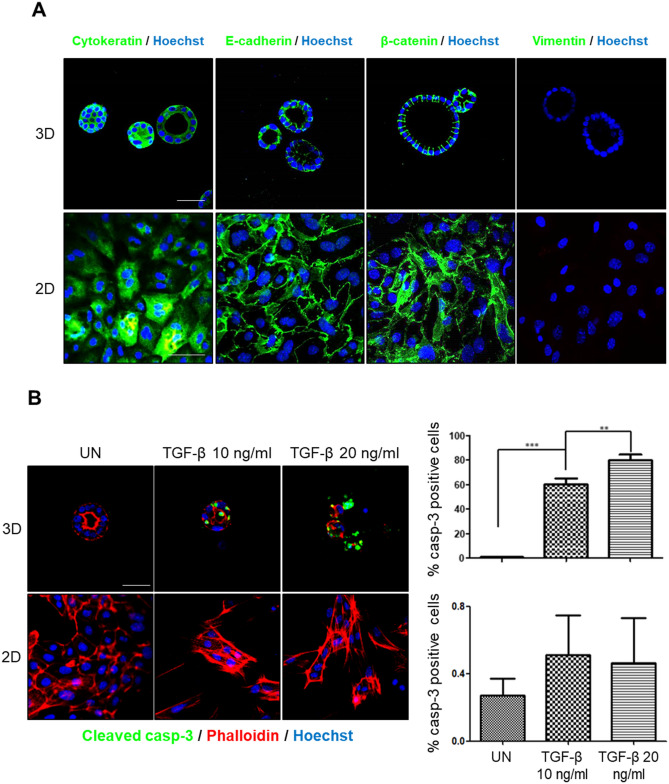
(**A**) Cytokeratin, E-cadherin, β-catenin and vimentin immunofluorescence on epithelial endometrial cells grown as 2D monolayers or 3D organoids show no differences in epithelial markers expression. Cells were counterstained with Hoechst to visualize nuclei. Scale bars: 25 µm. (**B**) Confocal images and quantification of cleaved caspase-3 immunofluorescence on endometrial cells grown as 2D monolayers or 3D organoids untreated (UN) or treated with 10 or 20 ng/ml TGF-β for 72 h. Cells were counterstained with phalloidin to visualize actin cytoskeleton and morphology and Hoechst to visualize nuclei. Scale bars: 25 µm. Values are mean and error bars represent mean ± S.E.M. **P < 0.01; ***P < 0.001 by one-way ANOVA, followed by the Tukey’s multiple comparison test.

We have previously demonstrated that TGF-β triggers apoptosis of polarized endometrial epithelial cells grown as organoids in 3D conditions^21^. Disruption of cell polarity is a hallmark of cancer and is intimately involved in cancer progression^6^. Therefore, we decided to analyze the effects of cellular polarization on pro-apoptotic response induced by TGF-β on endometrial epithelial cells. For this purpose, isolated endometrial cells were plated in presence or absence of Matrigel. As we have previously reported, Matrigel elicits a three-dimensional organization of endometrial cells into 3D organoids in which endometrial epithelial cells display a high grade of polarization^20^. In contrast, lack of Matrigel resulted in a conventional 2D monolayer endometrial cells culture. Although endometrial epithelial cells in 2D retained the expression of epithelial markers such as E-cadherin, cytokeratin or β-catenin and were negative for the mesenchymal marker vimentin, they did not show polarized morphology (Fig. 1A). To address the effects of Matrigel-induced cell polarization on TGF-β responses, both endometrial organoids and endometrial cell monolayers were treated with TGF-β. As we previously demonstrated, addition of TGF-β on endometrial cells cultured as 3D organoids caused a massive increase of cells displaying activation of caspase-3^21^. Surprisingly, treatment of 2D monolayers of epithelial endometrial cells lacking Matrigel with TGF-β did not lead to caspase-3 activation (Fig. 1B), indicating that lack of ECM impairs TGF-β-induced apoptosis.

### Lack of ECM (Matrigel) increases PI3K/AKT signaling which causes resistance to TGF-β-induced apoptosis

**Figure 2:**
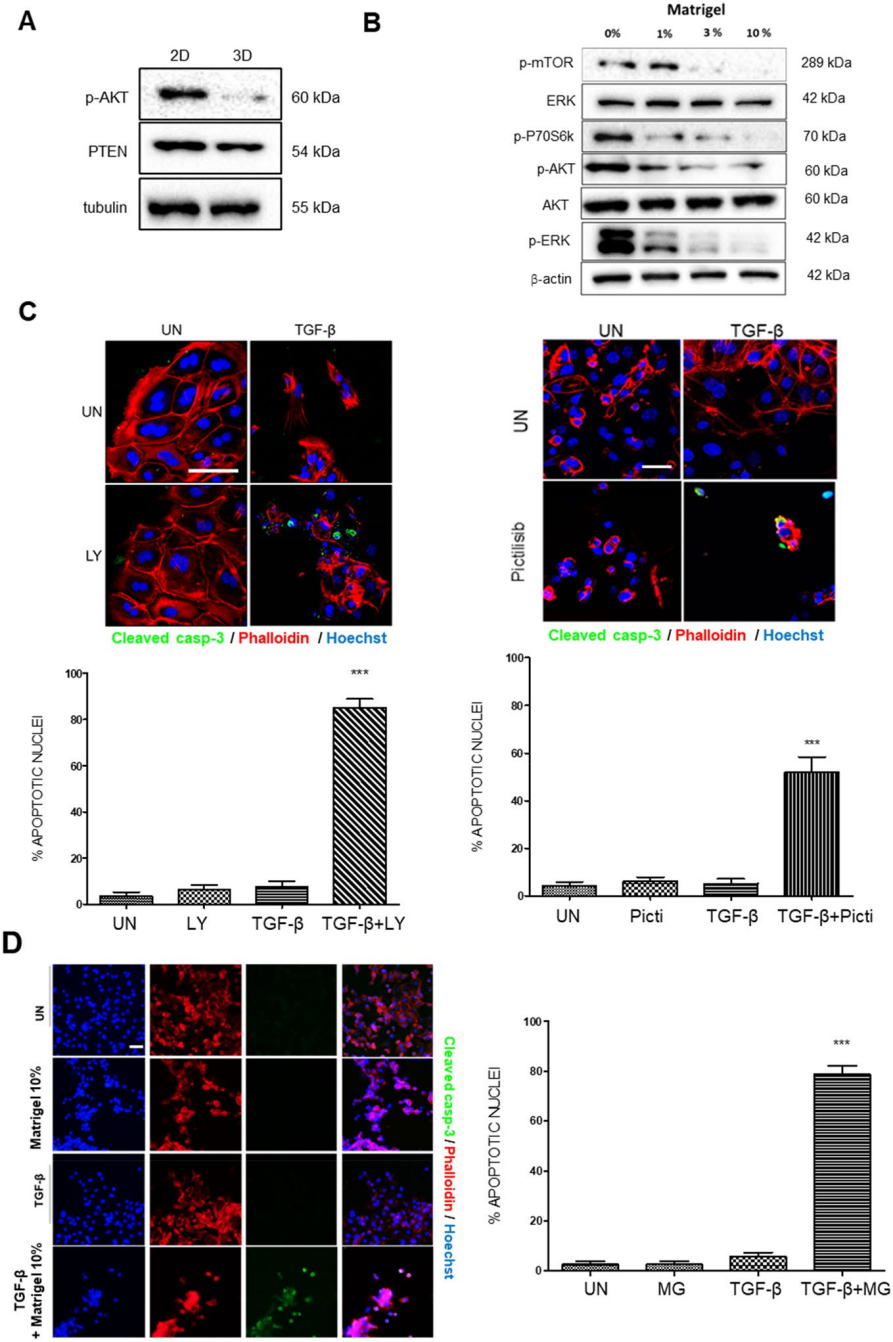
(**A**) Western blot analysis of phosphorylated AKT (p-AKT) and PTEN on lysates obtained from 2D monolayer or 3D organoid cultures. Membrane was re-blotted with tubulin to check for equal protein loading. (**B**) Western blot analysis of phosphorylated mTOR (p-mTOR), phosphorylated AKT (p-AKT), phosphorylated p70S6K, phosphorylated ERK, and PTEN on lysates obtained from 2D monolayer stimulated for 48 h with the indicated percentage of Matrigel diluted in basal culture medium. Membrane was re-blotted with β-actin, total ERK and total AKT to check for equal protein loading. (**C**) Representative confocal images and quantification of cleaved-caspase-3 immunofluorescence corresponding to 2D monolayer cultures with PI3K inhibitors. 20 µM LY294002 (LY) (left) or 200 nM Pictilisib (Picti) (Right). The cultures were treated with 20 ng/ml TGF-β alone, 20 µM LY, 200 nM Picti, combination of 20 ng/ml TGF-β plus 20 µM LY (TGF-β + LY) or 20 ng/ml TGF-β plus 200 nM Picti for 48 h. UN, untreated. Cells were counterstained with phalloidin to visualize actin cytoskeleton and morphology, and Hoechst to visualize nuclei. Scale bars: 25 µm. Values are mean and error bars represent mean ± S.E.M. ***P < 0.001 by one-way ANOVA, followed by the Tukey’s multiple comparison test. (**D**) Representative confocal images and quantification of cleaved-caspase-3 immunofluorescence corresponding to 2D monolayer cultures treated with TGF-β, 10% Matrigel, 10%Matrigel plus TGF-β or left untreated (UN). Cells were counterstained with phalloidin to visualize actin cytoskeleton and morphology, and Hoechst to visualize nuclei. Scale bars: 25 µm. Values are mean and error bars represent mean ± S.E.M. ***P < 0.001 by one-way ANOVA, followed by the Tukey’s multiple comparison test.

**Figure 3:**
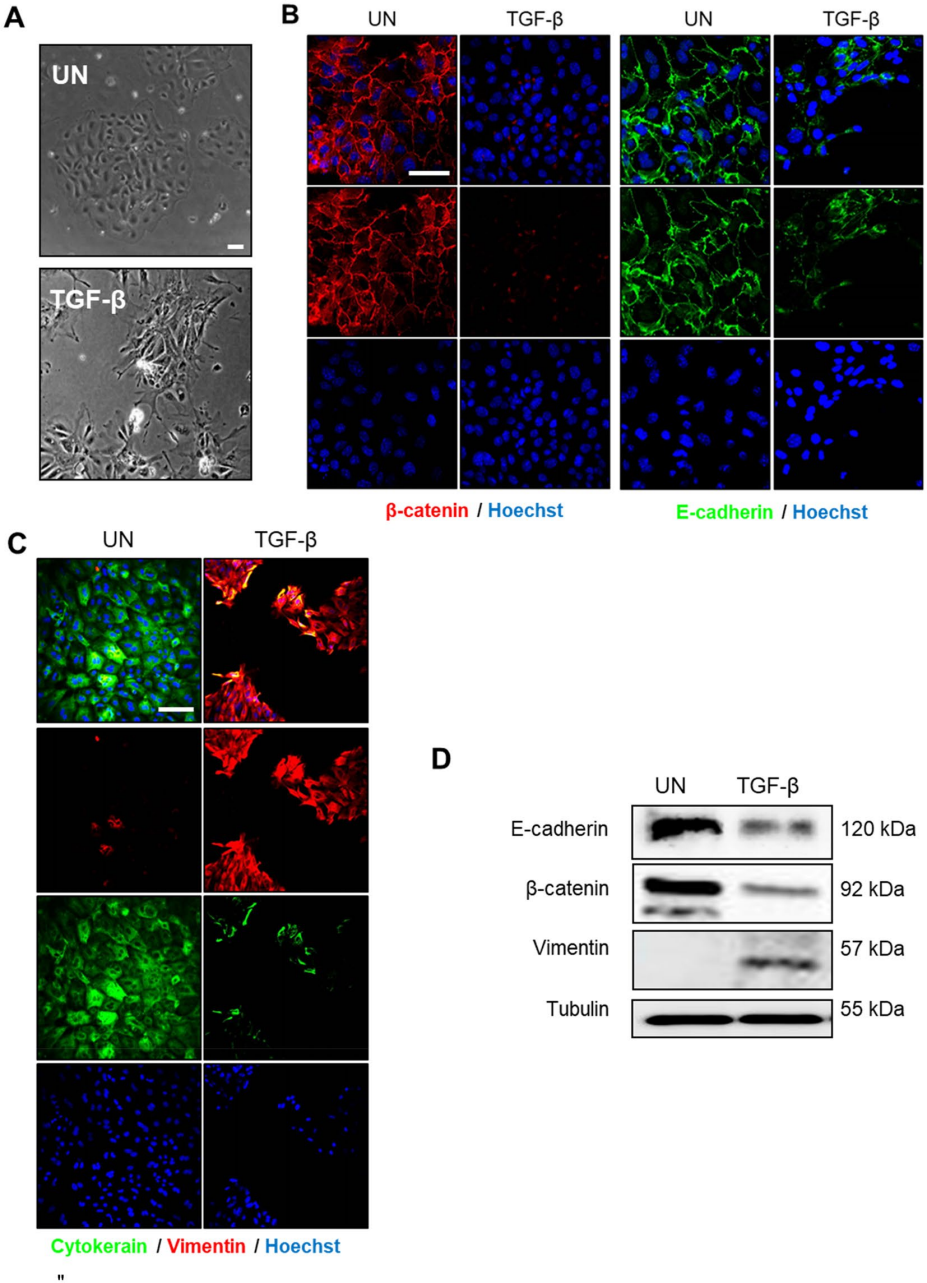
(**A**) Phase contrast images of 2D endometrial epithelial monolayers untreated (UN) or treated for 72 h with TGF-β 20 ng/ml. (**B**) Representative confocal images of β-catenin and E-cadherin immunofluorescences corresponding to 2D endometrial epithelial monolayers untreated (UN) or treated for 72 h with TGF-β 20 ng/ml. Cells were counterstained with Hoechst to visualize nuclei. Scale bars: 25 µm. (**C**) Representative confocal images of double cytokeratin and vimentin immunofluorescence of 2D endometrial epithelial monolayers untreated (UN) or treated for 72 h with TGF-β 20 ng/ml. Cells were counterstained with Hoechst to visualize nuclei. Scale bars: 25 µm. (**D**) Western blot analysis of E-cadherin, β-catenin and vimentin expression on lysates obtained from 2D monolayer stimulated for 72 h with TGF-β 20 ng/ml. Membrane was re-blotted with tubulin to check for equal protein loading.

The PI3K/AKT signaling pathway plays a pivotal role in the regulation of cell survival and apoptosis of endometrial epithelial cells. Our previous studies demonstrate that PTEN deficiency leads to increased PI3K/AKT signaling which hampers TGF-β-induced apoptosis of endometrial organoids^21,22^.These results enabled us to investigate whether ECM may regulate PI3K/AKT signaling pathway activation and thus sensibility to TGF-β-induced apoptosis. To address this hypothesis, AKT phosphorylation was analyzed by western blotting on 2D and 3D organoid lysates. As shown in Figs. 2A, 3D organoids displayed a markedly reduced level of AKT phosphorylation compared to 2D monolayers with no difference in PTEN expression. Next, we wondered whether such a difference in AKT phosphorylation could be the result of Matrigel stimulation itself or, otherwise, be the result of the acquisition of polarized glandular structure. To address this question, 2D monolayers of non-polarized endometrial epithelial cells were stimulated with increasing doses of Matrigel for 48 h and cell lysates were subjected to western blot analysis. Increasing of Matrigel concentration caused a marked reduction of mTOR, AKT and p70S6K phosphorylation with no changes in PTEN expression (Fig. 2B).

Having demonstrated that the absence of Matrigel results in increased AKT phosphorylation, we investigated whether such an increase was the cause of resistance to TGF-β induced apoptosis in 2D monolayers of endometrial cells. For this purpose, 2D monolayers were pre-treated with the PI3K inhibitors LY294002 or Pictisilib and then stimulated with TGF-β. Quantification of cleaved caspase-3 immunofluorescence revealed that inhibition of PI3K/AKT with both inhibitors restored TGF-β-induced apoptosis in 2D monolayers of endometrial epithelial cells (Fig. 2C). We performed western blot analysis of matched cultures to demonstrate that LY294002 and Pictisilib were inhibiting AKT phosphorylation (Supplementary Figure 1).

Having demonstrated that 10% Matrigel causes a decrease of AKT phosphorylation (Fig. 2B) and that PI3K/AKT inhibition causes TGF-β-induced apoptosis (Fiugre 2C), it is easy to speculate that Matrigel stimulation would also cause TGF-β-induced apoptosis. Quantification of cleaved caspase-3 immunofluorescence revealed that addition of 10% Matrigel caused TGF-β-induced apoptosis (Fig. 2D).

### TGF-β induces EMT-like changes in non-polarized 2D monolayers of epithelial cells

Phase-contrast observation of non-polarized cells treated TGF-β for 72 h evidenced a significant change in cell morphology, with transition from typical cobblestones morphology to mesenchymal spindle-shaped and fusiform feature (Fig. 3A). Since TGF-β is also a potent EMT inducer^15,23^, we intended to investigate whether such TGF-β morphological changes were the result of an EMT process. To address this point, we performed an immunofluorescence analysis for the epithelial markers cytokeratin, E-cadherin and β-catenin and for the mesenchymal marker vimentin. Treatment of non-polarized 2D endometrial cells with TGF-β resulted in loss of expression of the epithelial markers E-cadherin, β-catenin (Fig. 3B) and cytokeratin (Fig. 3C) and the acquisition of the mesenchymal marker expression vimentin (Fig. 3C), suggesting that endometrial cells were undergoing EMT. The results obtained by immunofluorescence analysis were further confirmed by western blot (Fig. 3D).

### Inhibition of ERK signaling blocks TGF-β-induced EMT in non-polarized 2D monolayers of endometrial epithelial cells

**Figure 4:**
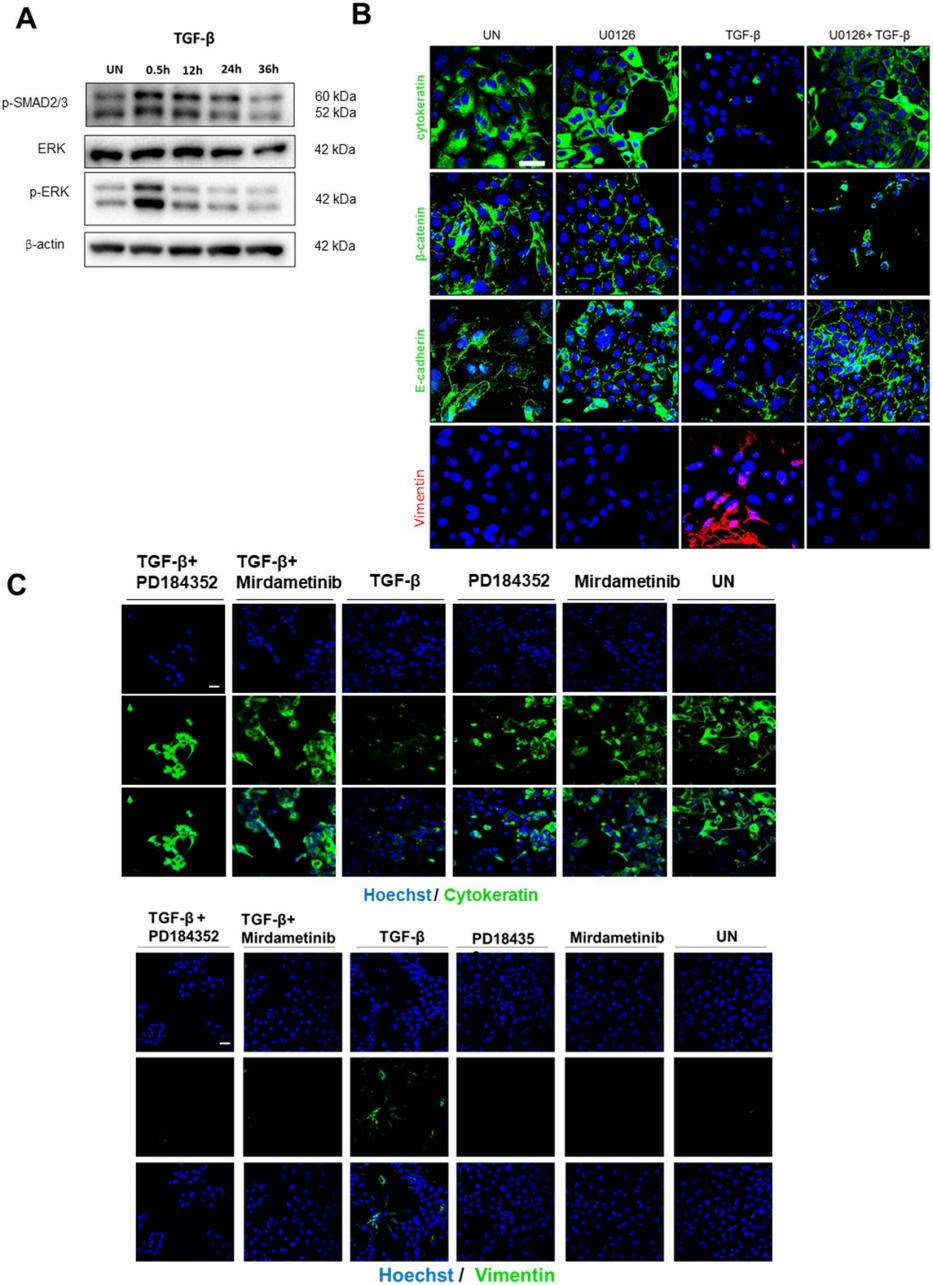
(**A**) Western blot analysis of phosphorylated ERK, PTEN on lysates obtained from 2D monolayer stimulated for the indicated times with TGF-β. Membrane was re-blotted with β-actin and total ERK to check for equal protein loading. (**B**) Representative confocal images of β-catenin, E-cadherin, cytokeratin and vimentin immunofluorescences corresponding to 2D endometrial epithelial monolayers untreated (UN) or treated for 72 h with TGF-β 20 ng/ml, U0126 10 µM (U0) or the combination (TGF-β + U0). Cells were counterstained with Hoechst to visualize nuclei. Scale bars: 25 µm. (**C**) Representative confocal images of cytokeratin and vimentin immunofluorescences corresponding to 2D endometrial epithelial monolayers untreated (UN) or treated for 72 h with TGF-β 20 ng/ml, PD184352 0.5 µM, Mirdametinib 20 nM or the combination their combination with TGF-β. Cells were counterstained with Hoechst to visualize nuclei. Scale bars: 25 µm.

Having demonstrated that treatment with TGF-β of non-polarized cells induced EMT, we sought to investigate the intracellular signaling pathways involved in EMT signal transduction following TGF-β treatment in non-polarized cells. We have previously demonstrated that ERK/MAPK is required for EMT of endometrial cancer cell lines transduced with a mutant BRAF-V600E mutant^24^. Such evidence enabled us to investigate the role of ERK in TGF-β-induced EMT. For this purpose, we first analyzed whether TGF-β was able to stimulate ERK activation. Western blot analysis of time course stimulation with TGF-β of endometrial cells, demonstrated an increase in ERK phosphorylation after 30 min of stimulations (Fig. 4A). Then, non-polarized 2D monolayers of endometrial cells were treated with TGF-β alone or with TGF-β plus the MEK inhibitor U0126 and the expression of EMT markers was analyzed by immunofluorescence. U0126 inhibited the loss of cytokeratin, E-cadherin or β-catenin expression and the increase of the mesenchymal marker vimentin (Fig. 4B). These results were further confirmed using two additional MEK inhibitors PD184352 and Mirdametinib and PD184352 (Fig. 4C). These results indicate that inhibition ERK signaling by MEK inhibitors prevented TGF-β-induced EMT. We also performed western blot analysis of matched cultures to demonstrate that MEK inhibitors were inhibiting ERK phosphorylation (Supplementary Figure 1).

### Matrigel blocks TGF-β-induced EMT in non-polarized 2D monolayers of endometrial epithelial cells

**Figure 5:**
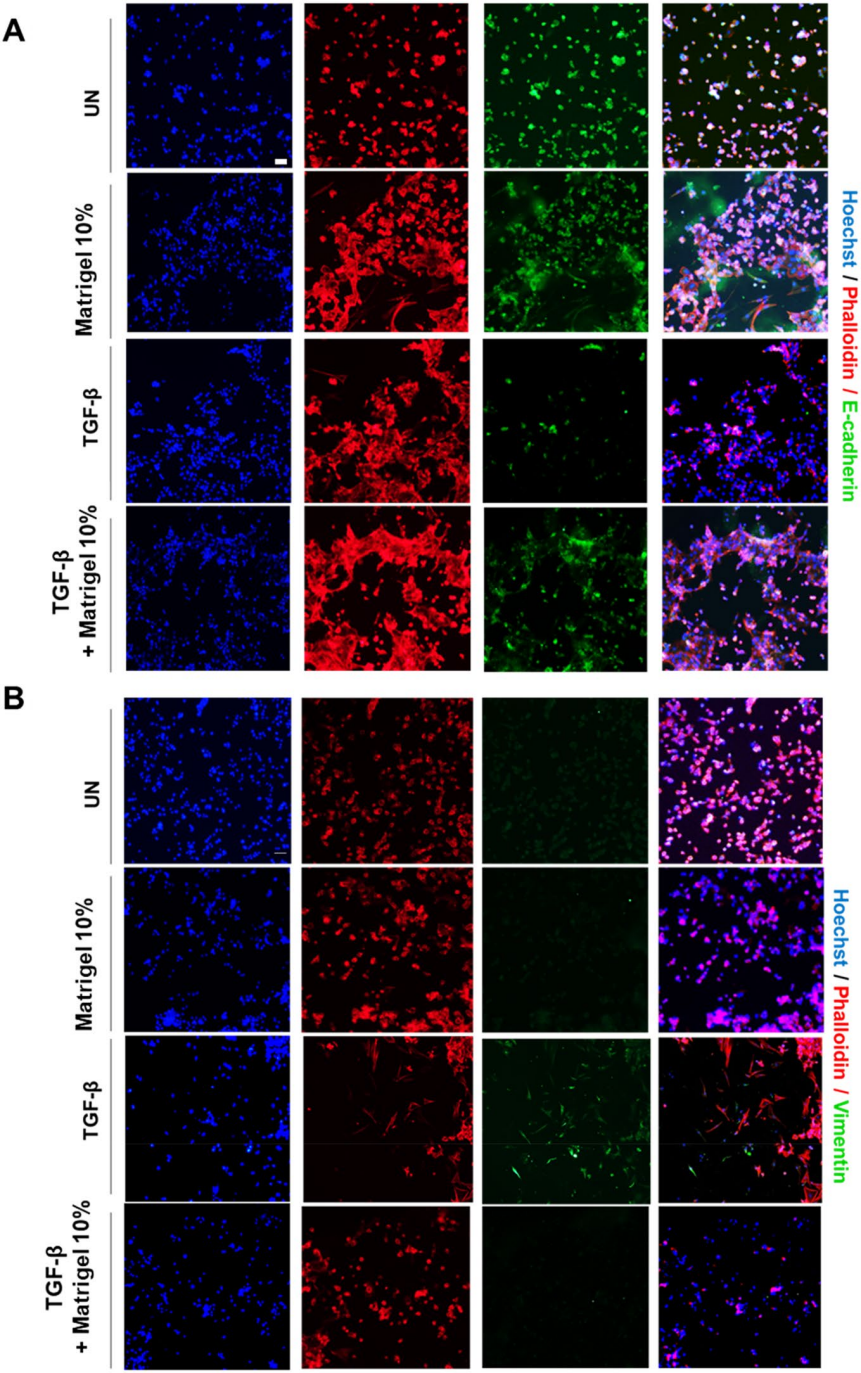
Representative confocal images of double phalloidin//E-cadherin (**A**) or phalloidin/Vimentin (**B**) immunofluorescence corresponding to 2D monolayer cultures treated with TGF-β, 10% Matrigel, 10% Matrigel plus TGF-β or left untreated (UN). Cells were counterstained with phalloidin to visualize actin cytoskeleton and morphology, and Hoechst to visualize nuclei. Scale bars: 25 µm.

The above results have demonstrated that, on the one hand, Matrigel caused a marked decrease in ERK phosphorylation (Fig. 4A) and, on the other hand, that MEK inhibition inhibited TGF-β-induced EMT (Fig. 4B). Therefore, it is tempting to speculate that Matrigel stimulation should also be able to block TGF-β-induced EMT. To test this hypothesis, non-polarized 2D monolayers of endometrial cells were treated with TGF-β alone or with TGF-β plus 10% Matrigel (which causes complete inhibition of ERK phosphorylation) and the expression of E-cadherin (Fig. 5A) and vimentin (Fig. 5B) was assessed by immunofluorescence. Matrigel stimulation caused blocked TGF-β-induced decrease of E-cadherin expression and increase of vimentin expression.

### SMAD2/3 deficiency leads to partial EMT independently of TGF-β stimulation but inhibits TGF-β-induced EMT

**Figure 6:**
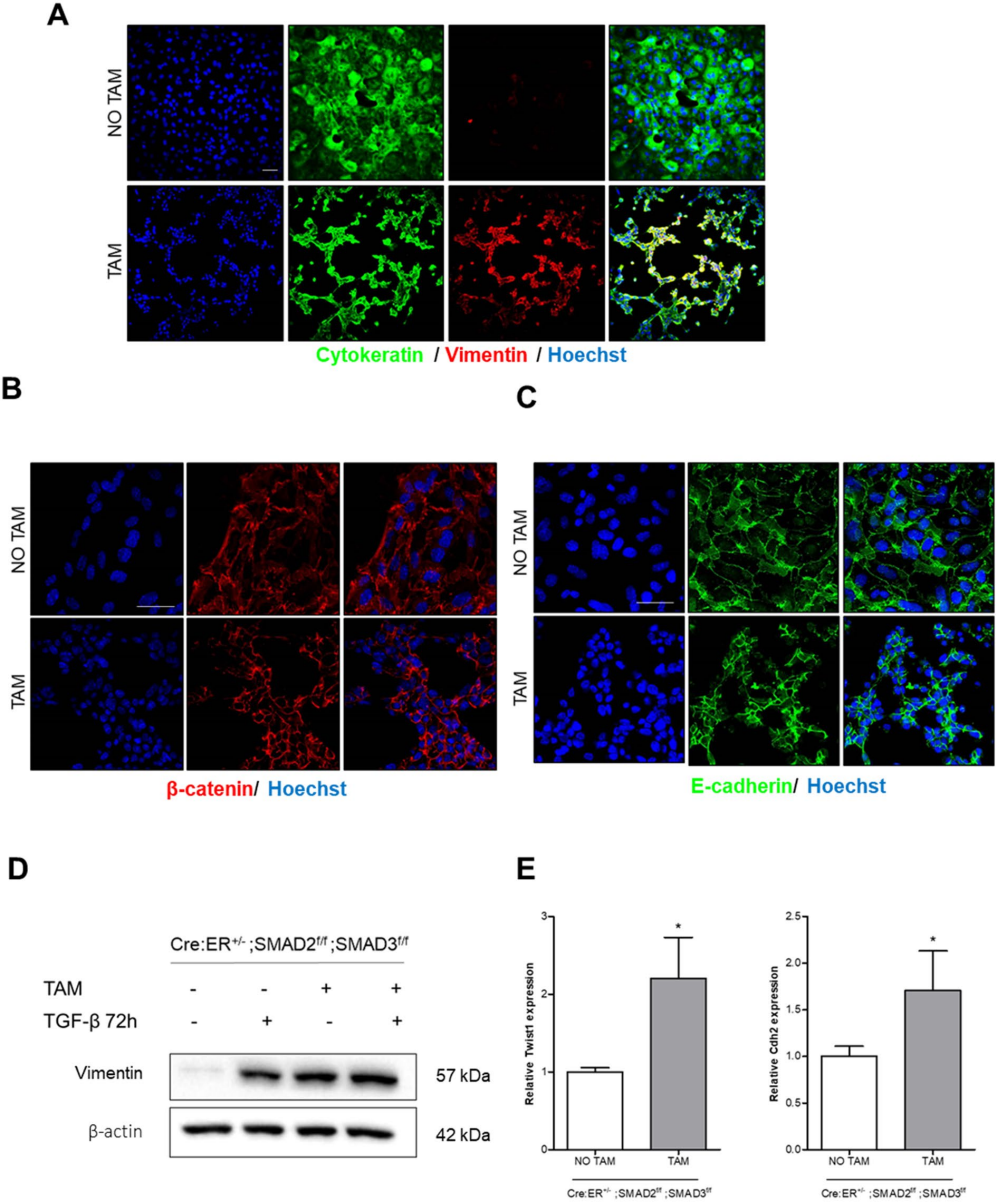
Representative confocal images of double cytokeratin and vimentin (**A**), β-catenin (**B**) or E-cadherin (**C**) immunofluorescence corresponding to 2D endometrial epithelial monolayers isolated from Cre:ER^+/−^; SMAD2^f/f^; SMAD3^f/f^, plated and treated with tamoxifen (TAM) to induce Cre:ER-mediated deletion of SMAD2 and SMAD3 alleles or left untreated (NO TAM). Images were captured after 3 days of tamoxifen-induced deletion. Cells were counterstained with Hoechst to visualize nuclei. Scale bars: 25 µm. **D.** Western blot analysis of vimentin expression on 2D endometrial epithelial monolayers isolated from Cre:ER^+/−^; SMAD2^f/f^; SMAD3^f/f^. Cells were treated (TAM) or not (NO TAM) with tamoxifen to induce Cre:ER-mediated deletion of SMAD2 and SMAD3 alleles and then stimulated or not with TGF-β for 72 h. Membranes were also blotted with β-actin antibody to ensure deletion of these proteins. **E.** RT-qPCR relative quantification of Cdh2 and Twist1 mRNA expression of 2D endometrial epithelial monolayers isolated from Cre:ER^+/−^; SMAD2^f/f^; SMAD3^f/f^. Cells were plated and treated (TAM) or not (NO TAM) with tamoxifen to induce Cre:ER-mediated deletion of SMAD2 and SMAD3 alleles. Values are mean and error bars represent mean ± S.E.M. *P < 0.001 by one-way ANOVA.

SMADs play an important role as transducers of cellular responses after TGF-β receptor engagement^11^. To address the role of these transcription factors in TGF-β-triggered EMT, we isolated endometrial epithelial cells from tamoxifen-inducible double SMAD2/3 knock-out mice (Cre:ER^+/−^; SMAD2^f/f^; SMAD3^f/f^). Isolated endometrial epithelial cells were plated in presence of tamoxifen to induce SMAD2/3 ablation and 24 h later cells were treated with TGF-β. Unexpectedly, SMAD2/3 deficient 2D endometrial cultures displayed a dramatic increase of vimentin expression in absence of TGF-β (Fig. 6A), while retaining the expression of epithelial markers such as cytokeratin (Fig. 6A), β-catenin (Fig. 6B) and E-cadherin (Fig. 6C). These results suggest that in the absence of ECM SMAD2/3 expression is required to maintain a full epithelial phenotype, and its loss causes partial EMT interpedently of TGF-β stimuli. The increase in vimentin expression was further validated by western blot. Noteworthy, the increase in vimentin protein levels of SMAD2/3 deficient cells was similar to those observed in wild type cells treated with TGF-β (Fig. 6D). To further confirm this process, we have analyzed the expression of other mesenchymal-replated markers by RT-qPCR. SMAD2/3 deficient cells displayed enhanced expression of Cdh2 (N-cadherin) and Twist1, one of the transcription factors involved in EMT (Fig. 6E).

**Figure 7:**
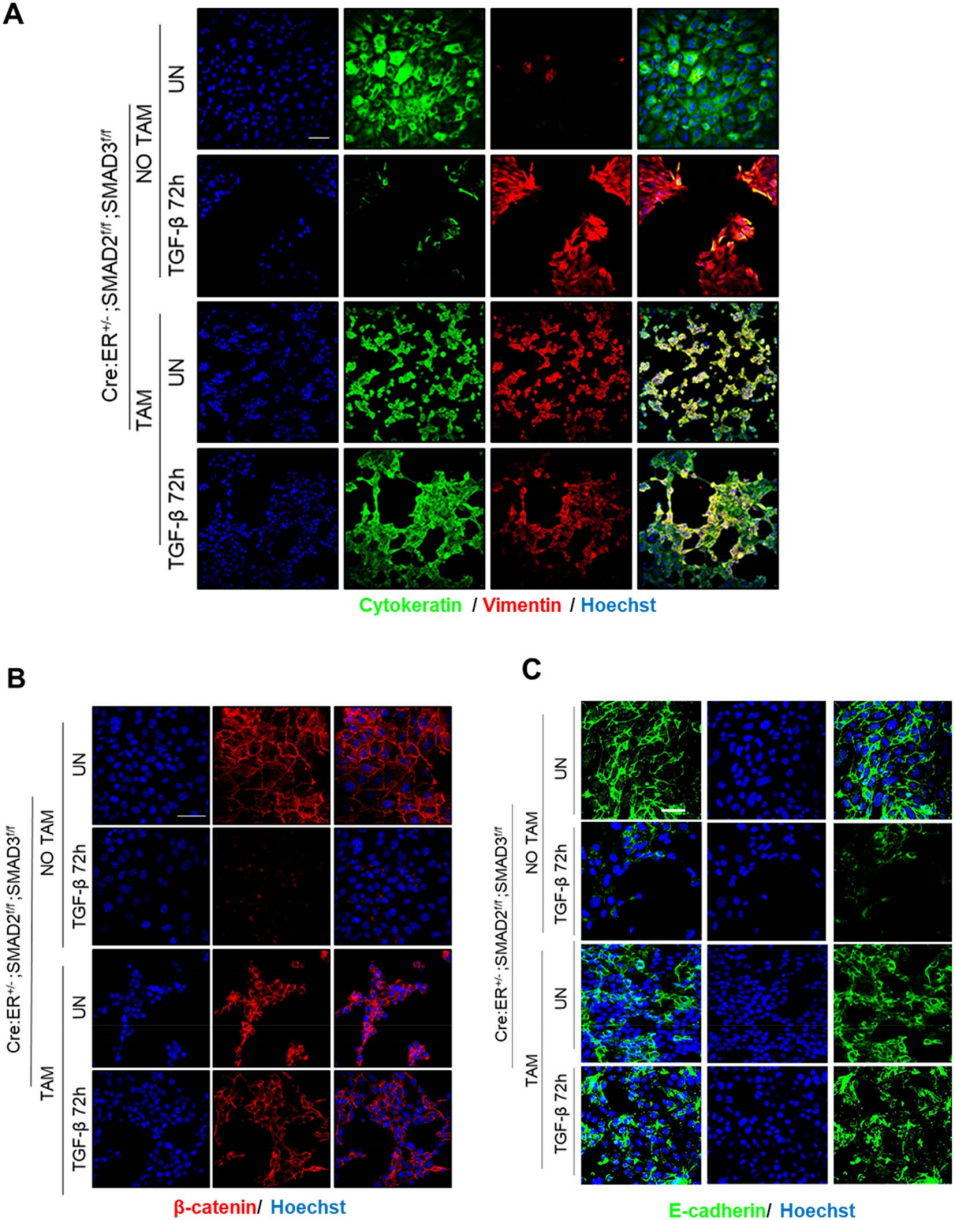
Representative confocal images of double cytokeratin and vimentin (**A**) β-catenin (**B**) or E-cadherin (**C**) immunofluorescence corresponding to 2D endometrial epithelial monolayers isolated from Cre:ER^+/−^; SMAD2^f/f^; SMAD3^f/f^. Cells were plated and treated with tamoxifen (TAM) to induce Cre:ER-mediated deletion of SMAD2 and SMAD3 alleles or left untreated (NO TAM) and then stimulated for 72 h with 20 ng/ml of TGF-β. Images were captured after 3 days of tamoxifen-induced deletion. Cells were counterstained with Hoechst to visualize nuclei. Scale bars: 25 µm.

Next, we analyzed the effect of SMAD2/3 deficiency on TGF-β-induced EMT. Proficient or SMAD2/3-deficient endometrial epithelial cells were treated with TGF-β 20 ng/ml for 72 h and the expression of vimentin and the epithelial markers β-catenin, cytokeratin and E-cadherin was assessed by immunofluorescence. In opposition to SMAD2/3 expressing cells, cells lacking SMAD2/3 did not show a significant reduction of epithelial markers cytokeratin (Fig. 7A), β-catenin (Fig. 7B) and E-cadherin (Fig. 7C). This result suggests that, in absence of ECM, SMAD2/3 deficiency blocks downregulation of epithelial cell markers related to TGF-β-triggered EMT changes.

### SMAD2/3 deficiency blocks the acquisition of migratory phenotype induced by TGF-β

**Figure 8:**
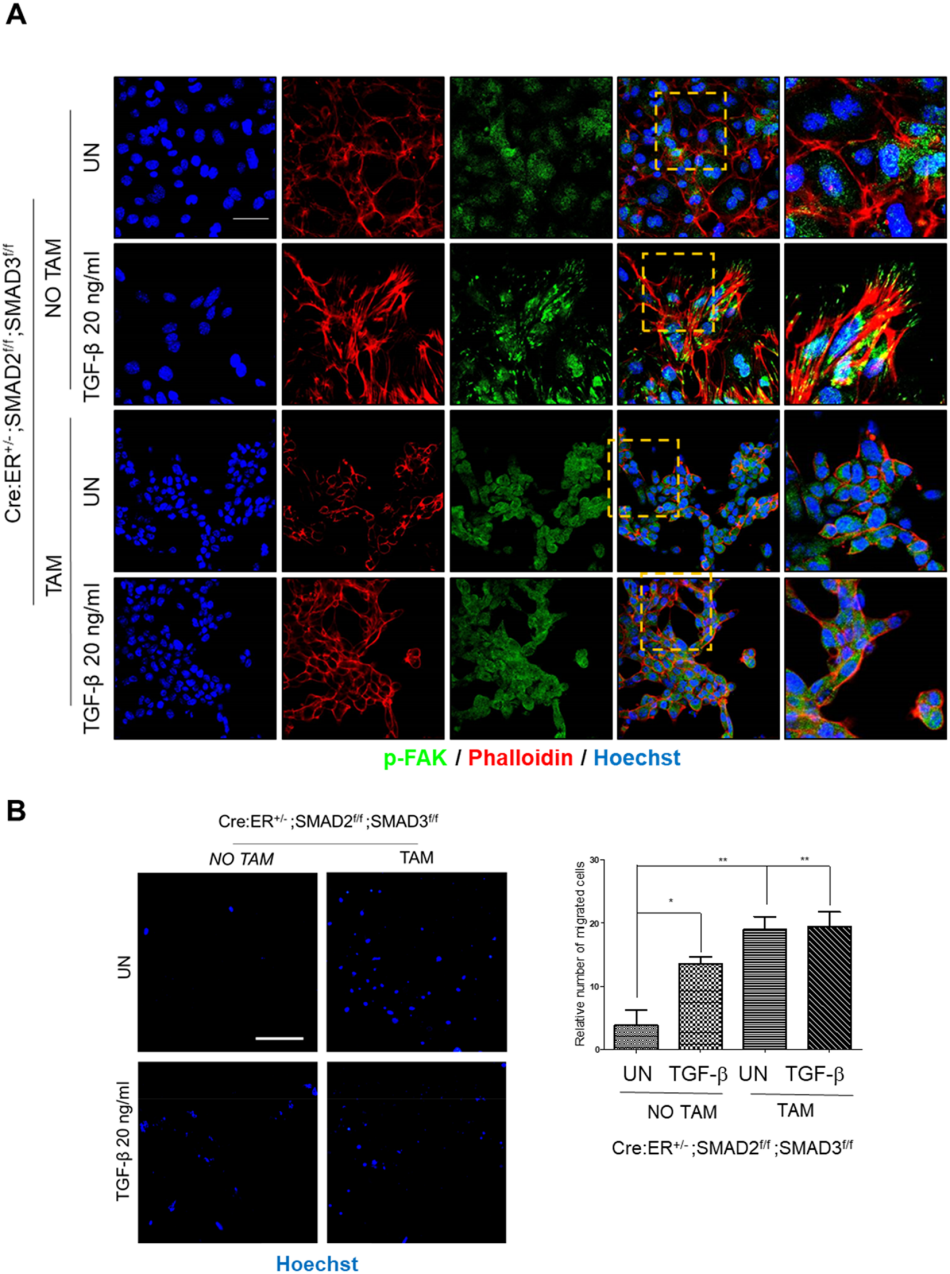
(**A**) Phalloidin and phosphorylated FAK (p-FAK) immunofluorescence corresponding to 2D endometrial epithelial monolayers isolated from Cre:ER^+/−^; SMAD2^f/f^; SMAD3^f/f^, plated and treated tamoxifen (TAM) to induce Cre:ER-mediated deletion of SMAD2 and SMAD3 alleles or left untreated (NO TAM) and then stimulated for 72 h with 20 ng/ml of TGF-β. Images were captured after 3 days of tamoxifen-induced deletion. Cells were counterstained with Hoechst to visualize nuclei. Scale bars: 25 µm. (**B**) Representative images of Hoechst staining images and quantifications of from Cre:ER^+/−^; SMAD2^f/f^; SMAD3^f/f^ transwell assay. Cells were plated on top of transwell inserts, treated with tamoxifen (TAM) or not (NO TAM) to induce SMAD2/3 deletion and then treated with TGF-β. After 72 h migrating cells were stained with Hoechst and quantified. Scale bars: 200 µm. Values are mean and error bars represent mean ± S.E.M. ***P < 0.001 by one-way ANOVA, followed by the Tukey’s multiple comparison test.

Once we analyzed the effects of SMAD2/3 deficiency on TGF-β-induced EMT markers expression, we investigated whether it may correlate with migratory capability of SMAD2/3-deficient cells. To address this issue, endometrial epithelial cells from Cre:ER^+/−^; SMAD2^f/f^; SMAD3^f/f^ were plated in presence of tamoxifen to induce SMAD2/3 ablation and 24 h later were treated with TGF-β. After TGF-β treatment, the morphology of actin cytoskeleton was evidenced by phalloidin staining and the phosphorylation of FAK by immunofluorescence. Phalloidin staining revealed that TGF-β induced stress fibers formation, indicative of migratory phenotype. TGF-β switched the scattered diffuse phospho-FAK staining to an increased punctuate staining pattern that co-localized with the spreading edge of the stress fibers. These staining patterns suggest that TGF-β triggers migration and formation of new focal adhesions of endometrial epithelial cells (Fig. 8A). In contrast, TGF-β treatment of endometrial cells lacking SMAD2/3 failed to change the pattern of phalloidin expression or phospho-FAK staining, indicating that deletion of SMAD2/3 impairs TGF-β-induced migration (Fig. 8A).

To further demonstrate increased migratory ability of SMAD2/3 knock-out cells, we performed a transwell migration assay. SMAD2/3 deficient cells displayed increased migratory capacity even in absence of TGF-β (Fig. 8B).

### ECM blocks increased vimentin expression caused by SMAD2/3 deficiency

**Figure 9:**
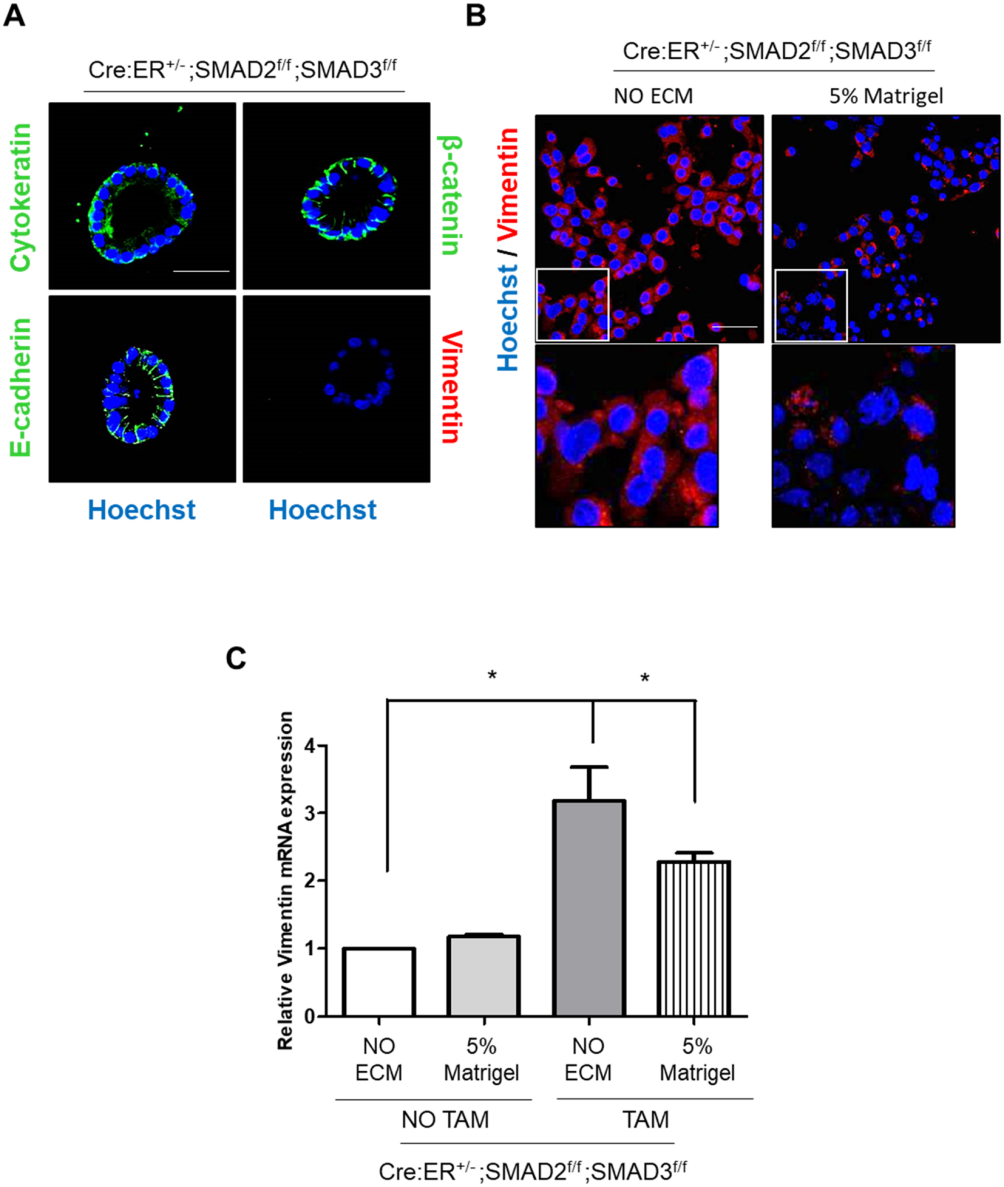
(**A**) Representative confocal images cytokeratin, E-cadherin, β-catenin and vimentin, on 3D organoid cultures from Cre:ER^+/−^; SMAD2^f/f^; SMAD3^f/f^ endometrial epithelial cells treated with tamoxifen to induce SMAD2/3 deletion. Scale bars: 25 µm. (**B**) Immunofluorescence corresponding to 2D endometrial epithelial monolayers isolated from Cre:ER^+/−^; SMAD2^f/f^; SMAD3^f/f^, plated and treated with tamoxifen (TAM) to induce Cre:ER-mediated deletion of SMAD2 and SMAD3 alleles then treated for 72 h with 5% Matrigel diluted in basal culture medium or left untreated (NO ECM). Images were captured after 3 days of tamoxifen-induced deletion. Cells were counterstained with Hoechst to visualize nuclei. Scale bars: 25 µm. (**C**) RT-qPCR relative quantification of vimentin mRNA expression of 2D endometrial epithelial monolayers isolated from Cre:ER^+/−^; SMAD2^f/f^; SMAD3^f/f^. Cells were plated and treated (TAM) or not (NO TAM) with tamoxifen to induce Cre:ER-mediated deletion of SMAD2 and SMAD3 alleles, then treated for 72 h with 5% Matrigel diluted in basal culture medium or left untreated (NO ECM). Results are expressed as mRNA expression relative to untreated (NO ECM). Values are mean ± S.E.M. *P < 0.01, by *t* test analysis.

Next, we investigated whether the partial EMT observed in 2D endometrial monolayers lacking SMAD2/3 was caused by the lack of ECM signals. For this purpose, we performed immunofluorescence analysis of vimentin and epithelial markers in SMAD2/3 deficient 3D organoid cultures. Three-dimensional organoids displayed positive staining for epithelial markers cytokeratin, β-catenin and absence of expression of vimentin, suggesting that ECM signals provided by Matrigel inhibit expression of vimentin (Fig. 9A). To further demonstrate that ECM is required to preserve the full epithelial phenotype in absence of SMAD2/3, 2D monolayers of SMAD2/3-deficient endometrial cells were acutely stimulated with 5% Matrigel diluted in culture medium and expression of vimentin was assessed by immunofluorescence and RT-qPCR. Indeed, Matrigel stimulation caused a marked reduction of vimentin immunostaining (Fig. 9B) and expression (Fig. 9C).

### ERK activation is required for SMAD2/3-induced vimentin expression

**Figure 10:**
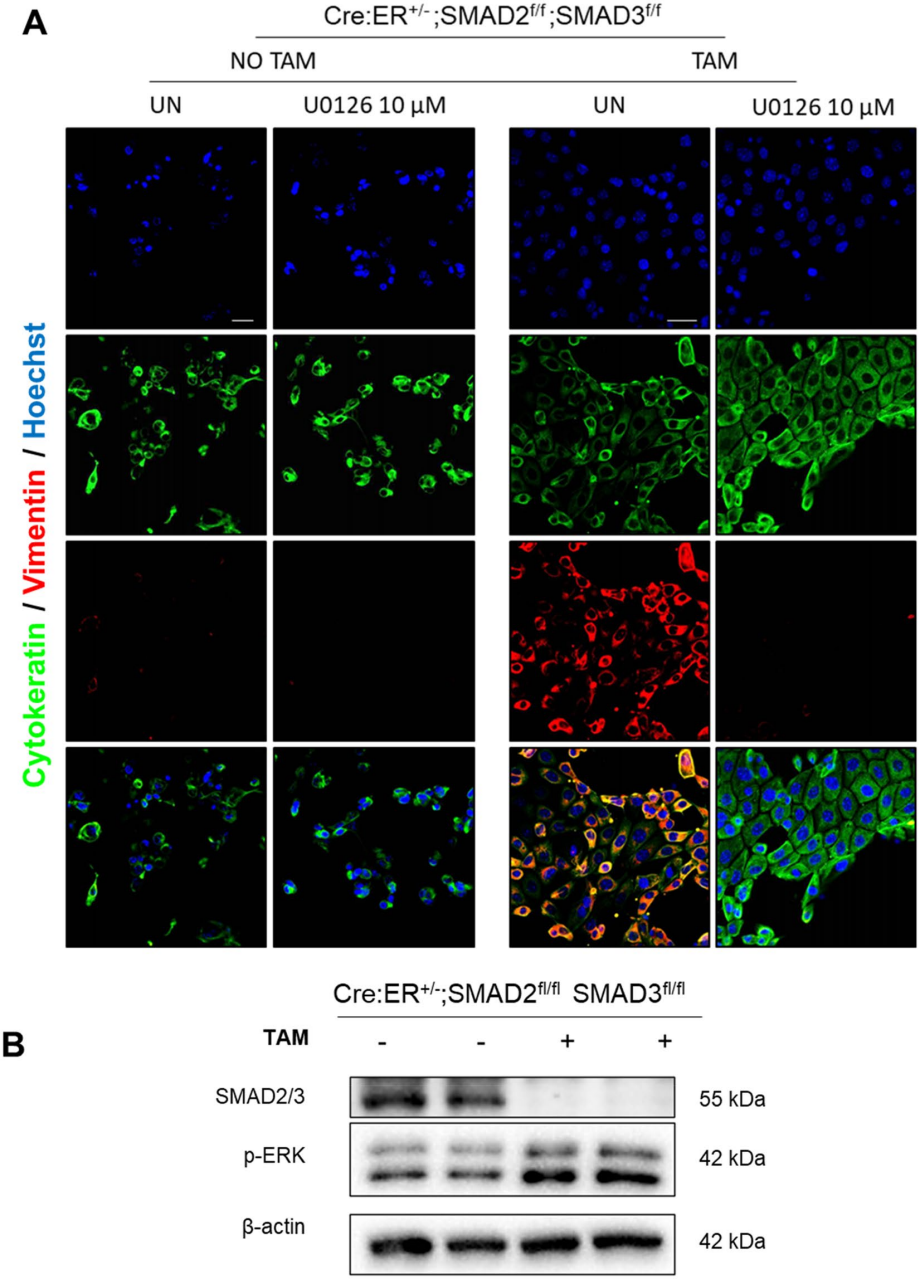
(**A**) Vimentin and cytokeratin immunofluorescence corresponding to 2D endometrial epithelial monolayers isolated from Cre:ER^+/−^; SMAD2^f/f^; SMAD3^f/f^, plated and treated with tamoxifen (TAM) to induce Cre:ER-mediated deletion of SMAD2 and SMAD3 alleles and treated with U0126 for 72 h. Cells were counterstained with Hoechst to visualize nuclei. Scale bars: 25 µm. (**B**) Western blot analysis of ERK phosphorylation on 2D endometrial epithelial monolayers isolated from Cre:ER^+/−^; SMAD2^f/f^; SMAD3^f/f^. Cells isolated from two different mice were plated and treated (TAM) or not (NO TAM) with tamoxifen to induce Cre:ER-mediated deletion of SMAD2 and SMAD3 alleles. Membranes were also blotted with SMAD2/3 antibodies to ensure deletion of these proteins.

The above presented results demonstrated that TGF-β-induced EMT-like changes require ERK activation, and, on the other hand, loss of SMAD2/3 resulted in acquisition of vimentin expression in unstimulated cells, but inhibited TGF-β-induced downregulation of epithelial markers. The next question we addressed was whether expression of vimentin in SMAD2/3-deficient endometrial cells was also reliant on ERK activation. For this purpose, endometrial epithelial cells isolated from tamoxifen-inducible double SMAD2/3 knock-out mice (Cre:ER^+/−^; SMAD2^f/f^; SMAD3^f/f^) were plated in presence of tamoxifen to induce SMAD2/3 ablation and 24 h later were treated with MEK inhibitor U0126 for further 72 h and the expression of EMT markers was analyzed by immunofluorescence. Treatment with U0126 resulted in completely loss of vimentin expression without affecting cytokeratin expression of endometrial cells lacking SMAD2/3 (Fig. 10A). This result indicates that ERK activation is also required for increased vimentin downstream of SMAD2/3 loss.

Having demonstrated that enhanced both TGF-β-induced ERK activity and that lack of SMAD2/3 increase vimentin expression, it is reasonable to think that loss of SMAD2/3 could cause and increase of basal ERK phosphorylation. To address this point, we performed western blot on lysates from two independent cultures from Cre:ER^+/−^; SMAD2^f/f^; SMAD3^f/f^ in which induced SMAD2 and SMAD3 deletion by tamoxifen addition. The results demonstrate that loss of SMAD2/3 expression leads to increased levels of ERK activity (Fig. 10B).

## Discussion

Knowledge of biological determinants deciding cellular responses after TGF-β stimulation are a complex and not completely resolved issue. In uterus, TGF-β plays an important role in its development, structure and function^25,26^. Genetically modified mouse models harboring alterations in TGF-β signaling elements have supported role of TGF-β in uterine physiology and pathology. Conditional deletion of TGF-βRI in the uterus leads to structural and functional defects in this organ^27^. Besides its role in uterine development, disruption of TGF-β signaling plays an important role in endometrial carcinogenesis^28,29^. As in other malignancies, TGF-β can play a dual function in endometrial cancer progression, acting as tumor suppressor in early stages and as a tumor promoter in later stages. Endometrial cancer display disabled TGF-β signaling^30^ leading to loss of growth inhibition^31^ acquisition of an invasive phenotype^32^ and correlates with poor prognosis^33^. Moreover, TGF-β has been shown to increase aggressiveness of endometrial cancer cell lines^32,34^. The role of TGF-β/SMAD pathway in endometrial carcinogenesis has also been revealed by conditional abrogation of TGF-β signaling elements through conditional deletion of TβRI^35^, conditional double deletion of SMAD2 and SMAD3^36^ or conditional deletion of TβRI in combination with PTEN-inactivated endometrium^37^. All of them result in metastatic endometrial carcinoma mice. In agreement with these results, we have previously demonstrated that TGF-β/SMAD signaling is an important tumor suppressive mechanism in mouse endometrial cells^21,22^. In the past, our laboratory established a 3D culture method to grow endometrial epithelial cells as organoids in presence of Matrigel as artificial ECM^20^. Using this approach, we demonstrated that TGF-β induces apoptosis of mouse 3D endometrial organoids, which is completely impaired by the genetic deletion of SMAD2 and SMAD3^21,22^. In the present work, we show that culture of endometrial epithelial cells without Matrigel as artificial ECM leads to growth of 2D traditional monolayers that retain epithelial phenotype, as indicated by immunofluorescence of epithelial and mesenchymal markers. However, treatment of these 2D cultures with TGF-β induced EMT-like changes instead of the apoptotic cell death observed in 3D organoids. These results support the hypothesis that polarized 3D epithelial organization could act as non-canonical tumor suppressor that prevents the manifestation of neoplastic features. Disruption of glandular structure, including loss of apicobasal polarity, is a hallmark of epithelial cancers. Several crucial cell-polarity proteins are recognized proto-oncogenes or tumor suppressors, and basic mechanisms of cell polarity are often objectives of oncogenic signaling pathways^6^. Among all the factors involved in correct establishment of cell polarity and glandular organization, ECM plays a pivotal role. It has been shown that it plays a critical role in carcinogenesis^38^ and in the regulation of TGF-β functions^39^. Our results suggest that the availability of ECM may be one of the determinants of cell context-dependent cellular outcome after exposure to TGF-β in normal endometrial epithelial cells. To this regard, ECM and cell polarity have also been shown to regulate cellular responses to TGF-β of endometrial cancer cell lines^40^. Our results support the hypothesis that polarized 3D epithelial organization acts as a non-canonical tumor suppressor that prevents the manifestation of neoplastic features such as EMT. More importantly, the ECM can be considered as one of the determinants that turns tumor suppressor functions of TGF-β to tumor promoter ones.

We have further investigated the mechanisms involved in ECM-mediated cell outcome change after TGF-β treatment. We have found that the absence of ECM enhances AKT and ERK phosphorylation with different functional consequences: increased AKT phosphorylation leads to inhibition of TGF-β induced apoptosis and increased ERK phosphorylation results in EMT. The PI3K/AKT plays a crucial role in the regulation of endometrial cell survival and apoptosis and, therefore, in endometrial carcinogenesis^41^. In fact, our previous results demonstrate that enhanced PI3K/AKT signaling caused by the loss of PTEN is sufficient to confer resistance of endometrial epithelial cells to TGF-β-induced apoptosis^21^. ERK signaling plays a central role in regulation of EMT in many tumoral types, including endometrial epithelial cells^42,43^. In endometrial cancers, we have previously demonstrated that enhanced ERK signaling is triggered by constitutively active BRAF mutations^24^. Although it is widely accepted that ECM impacts on intracellular signaling^44^, its functional consequences are still controversial. On the one hand, ECM-activated intracellular pathways such as PI3K/AKT and RAS/ERK have been associated with increased cell survival, proliferation, and anoikis suppression. On the other hand, ECM-induced acquisition of cell polarity acts as non-canonical tumor suppressor mechanism^6^. Our findings demonstrate that the presence of ECM and acquisition of cell polarity determines TGF-β responses, but it can also be important for cancer cell drug sensitivity. For instance, the development of 3D cultures of colorectal cancer cell lines reduced both ERK and AKT phosphorylation, and enhanced sensibility to anti-cancer drugs^45^. Our results support a tumor suppressor function for ECM, as its absence results in apoptosis resistance and EMT, both hallmarks of tumoral phenotype.

Another issue that deserves discussion is the role of SMAD signaling on ECM-regulated TGF-β responses. Our previous results demonstrated that loss of SMAD2/3 completely impairs TGF-β-induced apoptosis of endometrial 3D organoids^21,22^. Here, we have demonstrated that deletion of SMAD2/3 also influences TGF-β-induced EMT on 2D monolayers. SMAD2/3 can play multiple and opposing TGF-β signaling functions depending of cellular context^8,9^.SMADs interact with other transcriptional regulators that influence on TGF-β-induced transcriptional program. These SMAD-interacting transcription factors are tissue-specific and can integrate inputs from other signaling pathways, thereby generating TGF-β versatility in the transcriptional response in context-dependent manner. On the one hand, our results demonstrate that loss of SMAD2/3 causes an increase in the expression of mesenchymal markers even in absence of TGF-β. On the other hand, SMAD2/3 deficiency completely blocks downregulation of epithelial markers, resulting in cells expressing both mesenchymal and epithelial markers. This phenomenon known as partial or hybrid EMT and has been received special attention because of its involvement in cancer metastasis and therapy resistance^19,46,47^. Accordingly, SMAD2/3 deficient cells displayed increased migratory capacity. Such hybrid phenotype caused by SMAD2/3 deficiency was suppressed by the inhibition of ERK signaling, indicating that ERK is required for acquisition of a full EMT phenotype Moreover, ERK phosphorylation was found to be increased in SMAD2/3 deficient cells, suggesting that its loss enhances ERK activity and, therefore, increased expression of mesenchymal markers.

In summary, our results demonstrate that ECM is one of the context factors that participates in the cellular responses to TGF-β in endometrial cells. Appropriate ECM-cell contacts can act as non-canonical tumor suppressor mechanism, which can collaborate with other traditional tumor suppressor mechanisms to maintain endometrial homeostasis.

## Supplementary Information

Supplementary Information 1.

Supplementary Information 2.

## Data Availability

The datasets analyzed during the current study are available from the corresponding author upon reasonable request.
