# Supplementary material for: Lack of extracellular matrix switches TGF-β induced apoptosis of endometrial cells to epithelial to mesenchymal transition
Source: Sci Rep. 2022 Sep 1;12:14821. doi: 10.1038/s41598-022-18976-1 (PMC9437059; doi:10.1038/s41598-022-18976-1)
Supplement: Supplementary file 1 — Supplementary Information 1. [file 41598_2022_18976_MOESM1_ESM.pptx]

## Slide 1
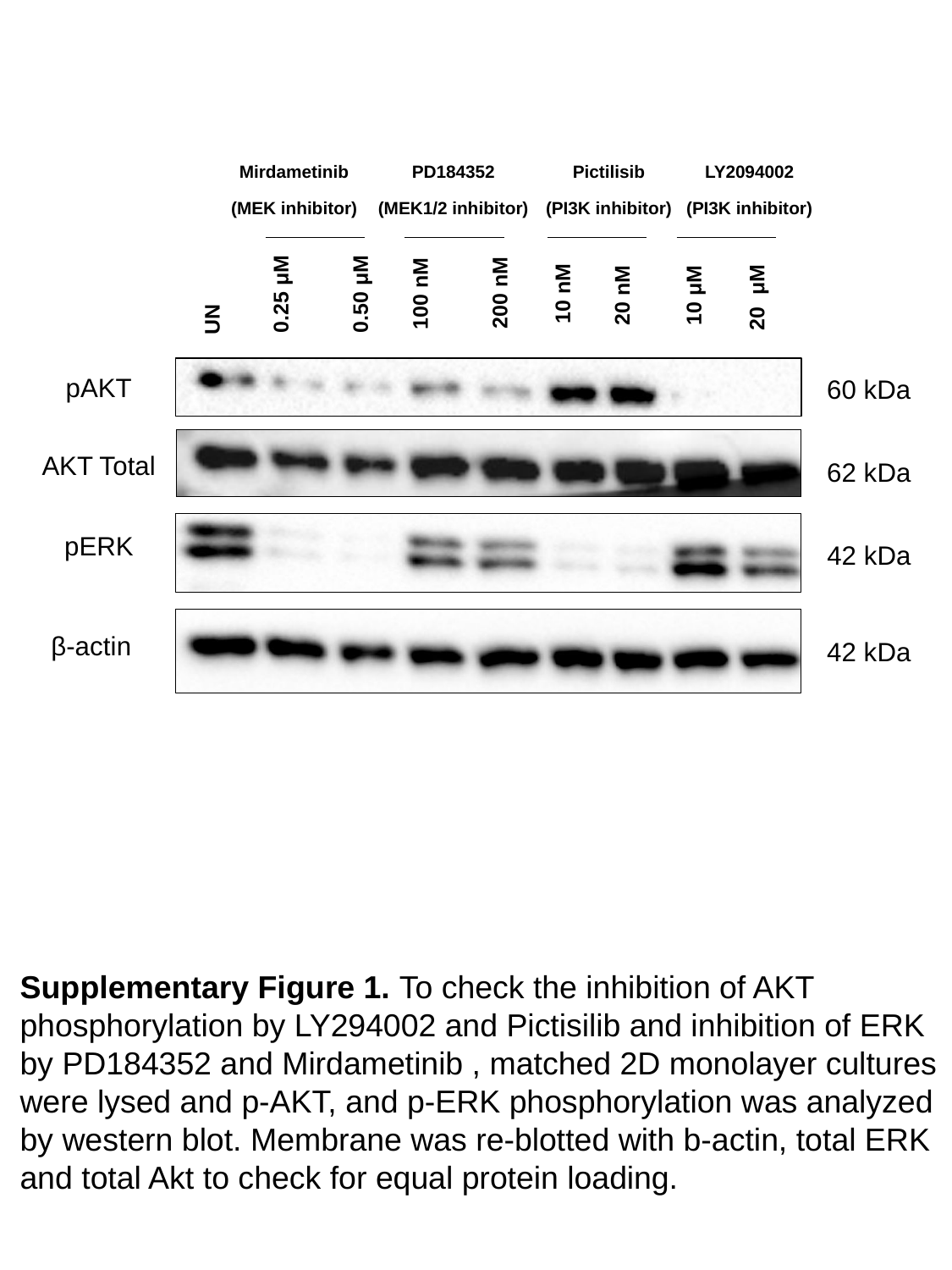

Mirdametinib
(MEK inhibitor)
PD184352
(MEK1/2 inhibitor)
Pictilisib
(PI3K inhibitor)
LY2094002
(PI3K inhibitor)
200 nM
0.25 μM
0.50 μM
100 nM
10 nM
20 nM
10 μM
20 μM
UN
60 kDa
pAKT
AKT Total
62 kDa
pERK
42 kDa
β-actin
42 kDa
Supplementary Figure 1. To check the inhibition of AKT phosphorylation by LY294002 and Pictisilib and inhibition of ERK by PD184352 and Mirdametinib , matched 2D monolayer cultures were lysed and p-AKT, and p-ERK phosphorylation was analyzed by western blot. Membrane was re-blotted with b-actin, total ERK and total Akt to check for equal protein loading.

## Slide 2
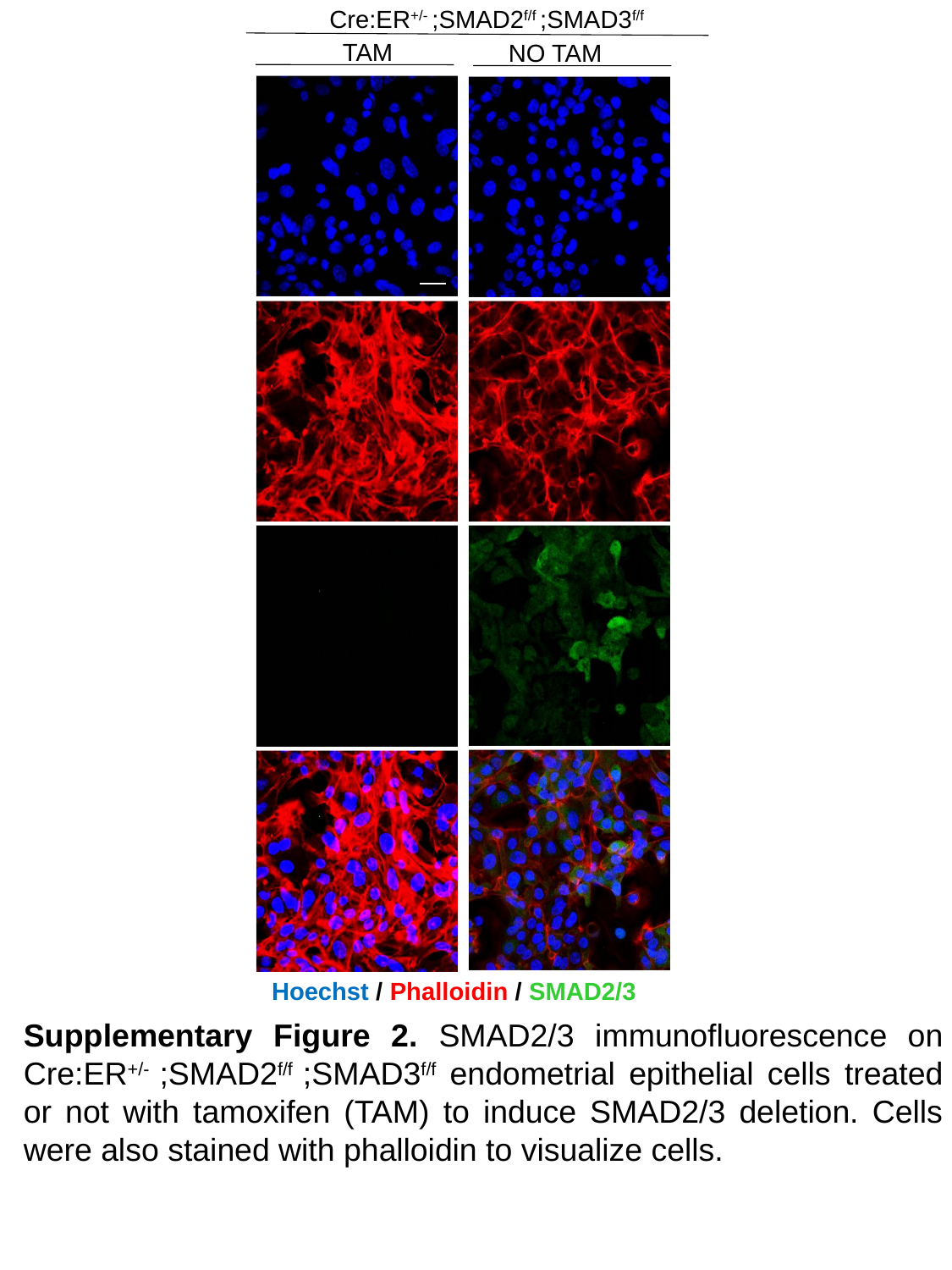

NO TAM
 Cre:ER+/- ;SMAD2f/f ;SMAD3f/f
Hoechst / Phalloidin / SMAD2/3
TAM
Supplementary Figure 2. SMAD2/3 immunofluorescence on Cre:ER+/- ;SMAD2f/f ;SMAD3f/f endometrial epithelial cells treated or not with tamoxifen (TAM) to induce SMAD2/3 deletion. Cells were also stained with phalloidin to visualize cells.
