# Supplementary material for: Lack of extracellular matrix switches TGF-β induced apoptosis of endometrial cells to epithelial to mesenchymal transition
Source: Sci Rep. 2022 Sep 1;12:14821. doi: 10.1038/s41598-022-18976-1 (PMC9437059; doi:10.1038/s41598-022-18976-1)
Supplement: Supplementary file 2 — Supplementary Information 2. [file 41598_2022_18976_MOESM2_ESM.pdf]

FIGURE 2A

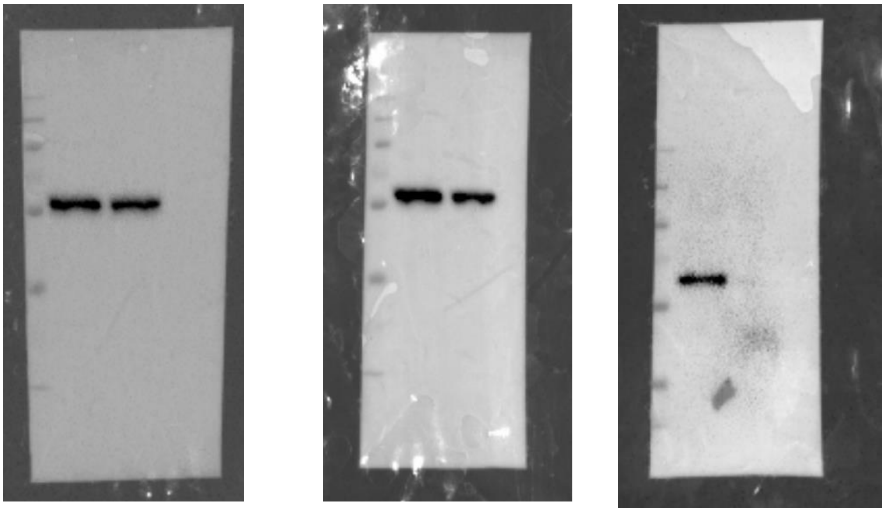

tubulin PTEN pAKT

FIGURE 2B

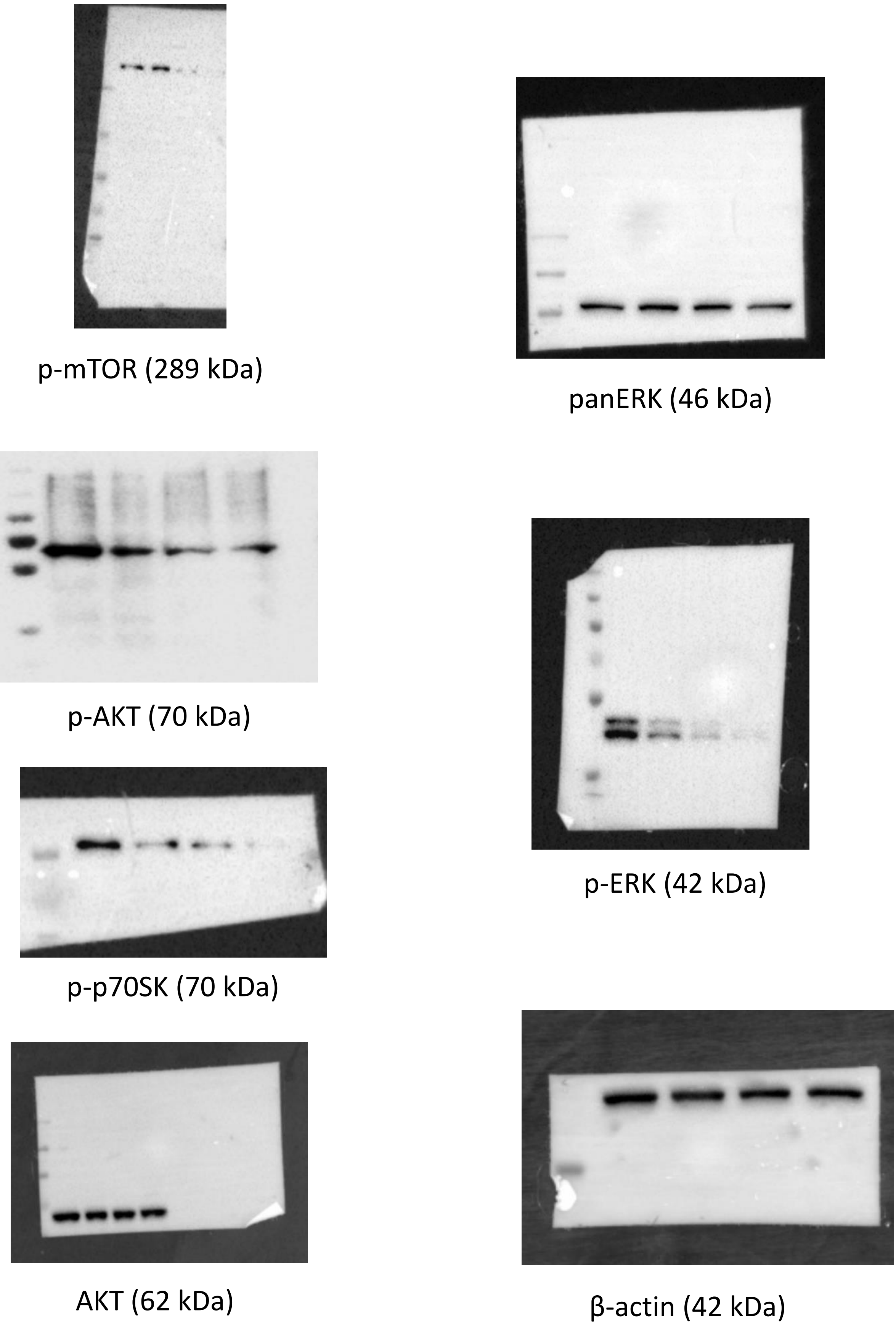

p-mTOR (289 kDa)
panERK (46 kDa)
p-AKT (70 kDa)
p-ERK (42 kDa)
p-p70SK (70 kDa)
AKT (62 kDa)
β-actin (42 kDa)

FIGURE 3D

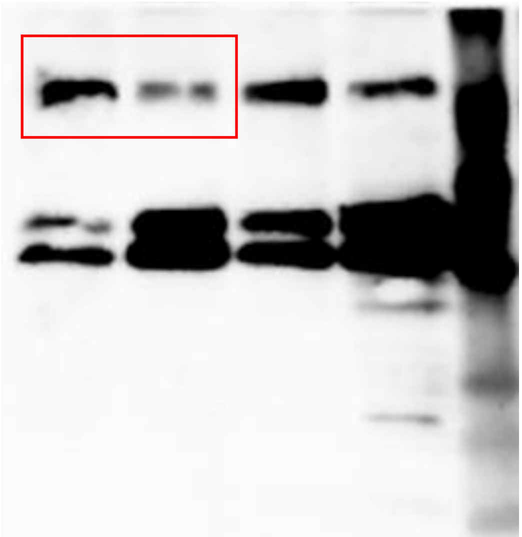

E-cadherin

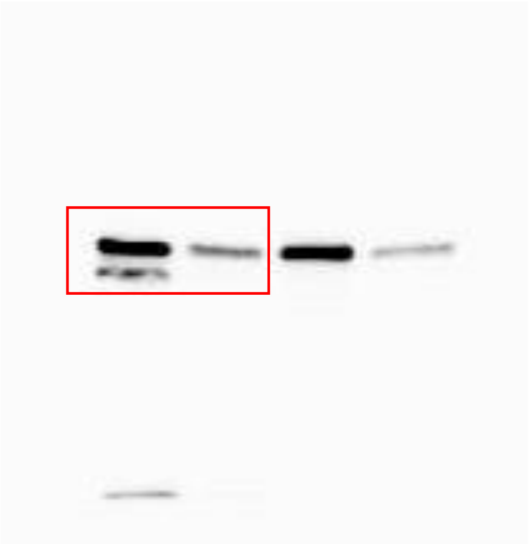

β-catenin

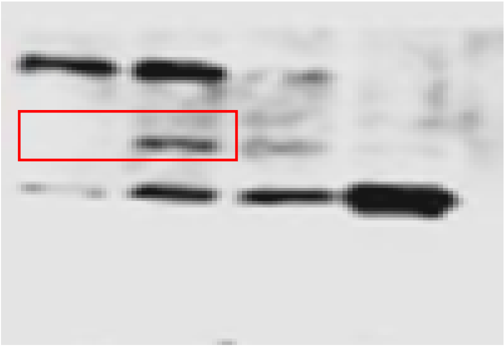

vimentin

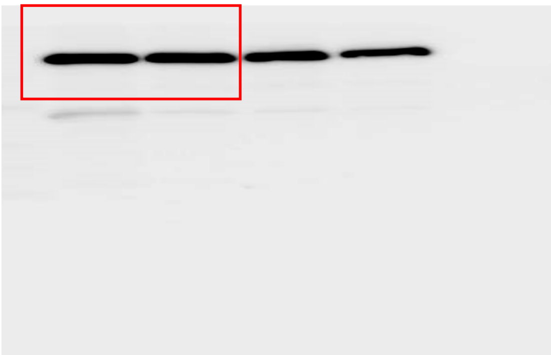

tubulin

FIGURE 4A

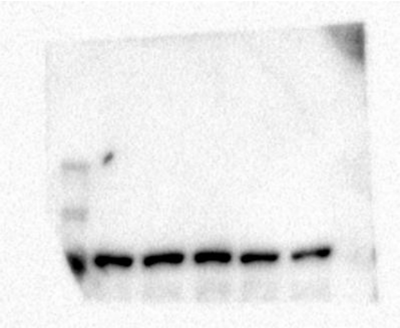

panERK (46 kDa)

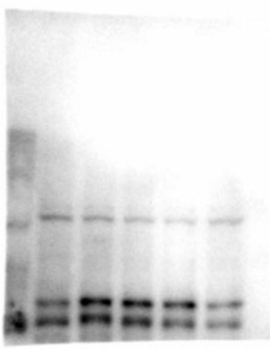

p-SMAD2-3 (60, 52 kDa)

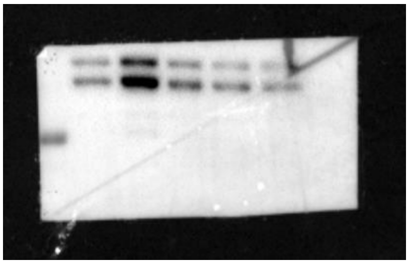

p-ERK (42 kDa)

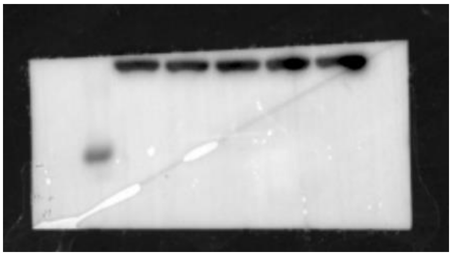

β-actin (42 kDa)

FIGURE 6D

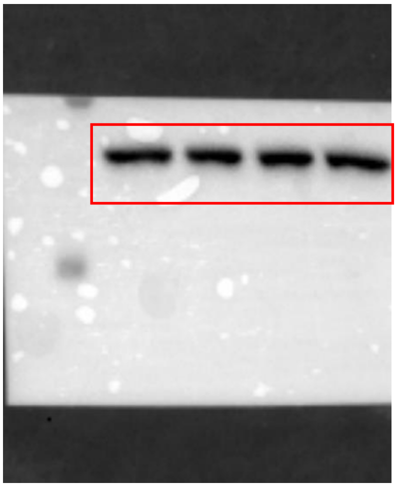

β-actin (42 kDa)

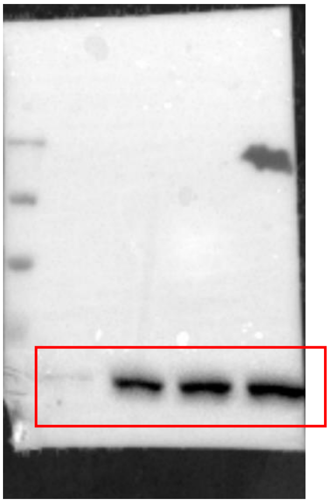

Vimentin (57 kDa)

FIGURE 10B

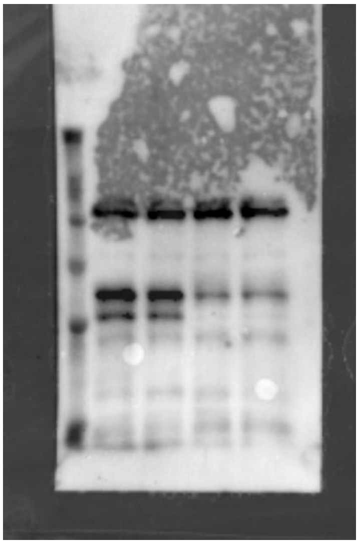

SMAD2/3 (55 kDa)

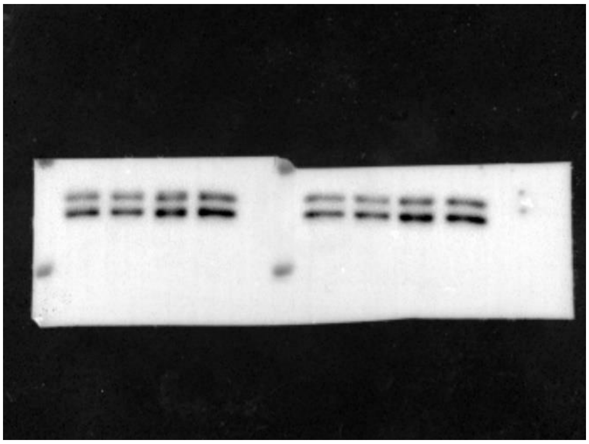

p-ERK (42 kDa)

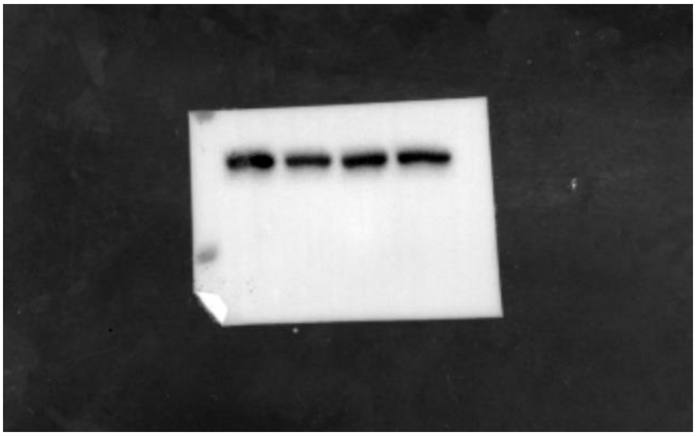

β-actin (42 kDa)
